# Genetically diverse populations hold the keys to climatic adaptation in the Western barn owl (*Tyto alba*)

**DOI:** 10.1038/s42003-026-10385-8

**Published:** 2026-06-03

**Authors:** Hugo Corval, Tristan Cumer, Alexandros Topaloudis, Flavien Collart, Antoine Guisan, Alexandre Roulin, Jérôme Goudet

**Affiliations:** 1 Department of Ecology and Evolution, University of Lausanne, Lausanne, Switzerland; 2 Swiss Institute of Bioinformatics, Lausanne, Switzerland; 3 Institute of Botany, University of Liège, Liège, Belgium

**Keywords:** Evolutionary genetics, Population genetics

## Abstract

Although local adaptation influences species distributions, its role in driving evolutionary resilience under climate change remains unclear. Current predictive models focus on genetic adaptation to present climates, providing limited insight into future adaptive capacity. We hypothesise that historical responses to climatic shifts can reveal candidate loci for local adaptation in the future. Combining ecological niche modelling and genomic analyses, we investigate spatiotemporal patterns and mechanisms of local adaptation of the Western Palearctic barn owl (*Tyto alba*). Ecological modelling reveals that barn owls now occupy a broader climatic niche than during the Last Glacial Maximum. Genomic analyses indicate ongoing adaptation, with regions under selection linked to environmental factors across all populations. We find that local adaptation drives evolutionary changes across populations, enabling colonisation of new habitats and shaping responses to climate change in resident populations. We show that standing genetic diversity plays a crucial role in adaptation to past, present, and future environmental shifts.

## Abstract (web summary)

Ecological niche modelling shows that Western barn owls occupy a broader climate niche than at the Last Glacial Maximum, while genomic analyses identify genetically diverse populations as key reservoirs for adaptation to climatic change.

## Introduction

Climatic variations affect biodiversity by impacting individual’s fitness, by driving population differentiation, and ultimately by shaping species distributions^1–3^. The extent of these impacts, however, depends on the speed and intensity of climatic variations^4^: sudden or extreme shifts can lead to local or global extinction if individuals fail to survive or reproduce^5^. In case of more gradual changes, the persistence of populations and species will depend on individuals’ ability to migrate or cope with their new environment^1,6,7^. Through migration, individuals track their suitable conditions in time and space to survive, inducing a shift in species distribution^1^. Individuals can also change physiologically, morphologically, or phenologically to cope with the new local conditions^7^. These changes can occur via phenotypic plasticity, the ability of individual genotypes to produce different phenotypes when exposed to various environmental conditions^8^ or through genetic adaptation, favouring different genotypes better adapted to the local ecological conditions^9^.

Considering the critical role of local adaptation in determining population persistence^10^, understanding whether individuals possess the intrinsic capacity to adapt to new environmental conditions is critical. In recent years, genomic offset has emerged as a key metric for predicting genetic maladaptation to future climates by linking environmental factors to allele frequencies and estimating the genetic changes needed for individuals to survive to new conditions^11–13^. However, while such predictions provide valuable insights into potential risks, they primarily project future offsets based on current genomic and environmental relationships, without incorporating standing genetic variation as an additional layer of information. Therefore, a key challenge remains understanding whether the existing genetic diversity can fuel adaptation to new climate, a question our study seeks to answer.

The end of the Last Glacial Maximum in the Western Palearctic created contrasting evolutionary contexts, offering a unique opportunity to study how climate change, along with neutral and selective processes, influenced local adaptation. In the north, temperatures and melting ice caps opened previously unsuitable regions to (re)-colonisation^2,14,15^. Populations that tracked their ancestral niche required little adaptation, whereas those entering novel environments likely experienced selection favouring individuals best suited to local conditions, promoting adaptation^9^. Yet, as colonisation advanced through repeated migration events, founder effects may have progressively eroded genetic diversity, constraining adaptive potential along the front of colonisation^16^. At the other end of the range, populations that persisted in the south also faced environmental shift, but their higher genetic diversity may have buffered them against change and enhanced their capacity to adapt^17^. Given these contrasting scenarios, where and how local adaptation happens remains elusive and addressing these questions can deepen our understanding of populations’ adaptive potential.

The barn owl (*Tyto alba*), a nonmigratory raptor distributed across the Western Palearctic, offers a powerful system to investigate the patterns and processes of local adaptation, a theme central to many species facing environmental heterogeneity. The species recolonised the Northern part of Europe at the end of the Last Glacial Maximum from two main glacial refugia located in the Iberian Peninsula and the Italian and Balkan Peninsulas^15^. Presently, its range stretches across the Western Palearctic^15^ where it faces heterogeneous climates. Despite a generally low level of genetic differentiation across its range, southern populations host a higher genetic diversity than northern populations^15,18,19^, and exhibit notable phenotypic variation, such as a cline in plumage coloration between southern and northern populations^18–21^. A previous study revealed that the colour cline cannot be explained by purely neutral processes and argued that selection for local adaptation was or is still acting on this phenotype^21^. The well-understood history of the barn owl in Europe since the Last Glacial Maximum, combined with the low genetic structure at the continental level and previous evidence of adaptive selection throughout its range, make this species an attractive model to test and quantify the extent and location of local adaptation in face of climatic variation, a question of relevance across many taxa.

Here, we evaluated where and how heterogeneous climate induce local adaptation using the European barn owl as a model organism. We first used ecological information and Species Distribution Modelling to (i) quantify the climatic heterogeneity faced by the barn owl nowadays and (ii) measure how these climatic conditions differ from those experienced during the Last Glacial Maximum. We then looked for an association between climatic variables and genomic variants from the entire genome of 74 owls from 9 different localities across Europe. We combined these results with a new approach to scan the genomes for traces of selection and identified genomic regions and genes potentially involved in the local adaptation of the different populations. We found a strong and common signal in southern populations. Overall, our results illustrate how the most diverse populations, often located at the core of the distribution, may host the candidate loci for local adaptation to face climate change, giving clues on how standing genetic variation can fuel local adaptation.

## Results

### Suitable conditions nowadays are more diverse than during the Last Glacial Maximum

Understanding the climatic context is fundamental for interpreting the evolutionary dynamics that have shaped both the population structure and adaptive responses of contemporary barn owl populations. The spatial prediction obtained by the species distribution model (SDM)^22^ highlights a striking increase of the area occupied by the barn owl since the Last Glacial Maximum^23^ (~ 20,000 years ago), similar to what was observed in trees^24^, mammals^25^, and birds^26^. During the Last Glacial Maximum, suitable habitats were mostly confined to regions around the Mediterranean, covering northern Africa as well as the Iberian, Italian, and Greek peninsulas (Supplementary Fig. 1). Today, favourable conditions extend well into Western, Central, and Northern Europe, making most of the continent suitable for barn owls. This notable northward and inland expansion prompted us to investigate the climatic variables driving these changes.

**Fig. 1:**
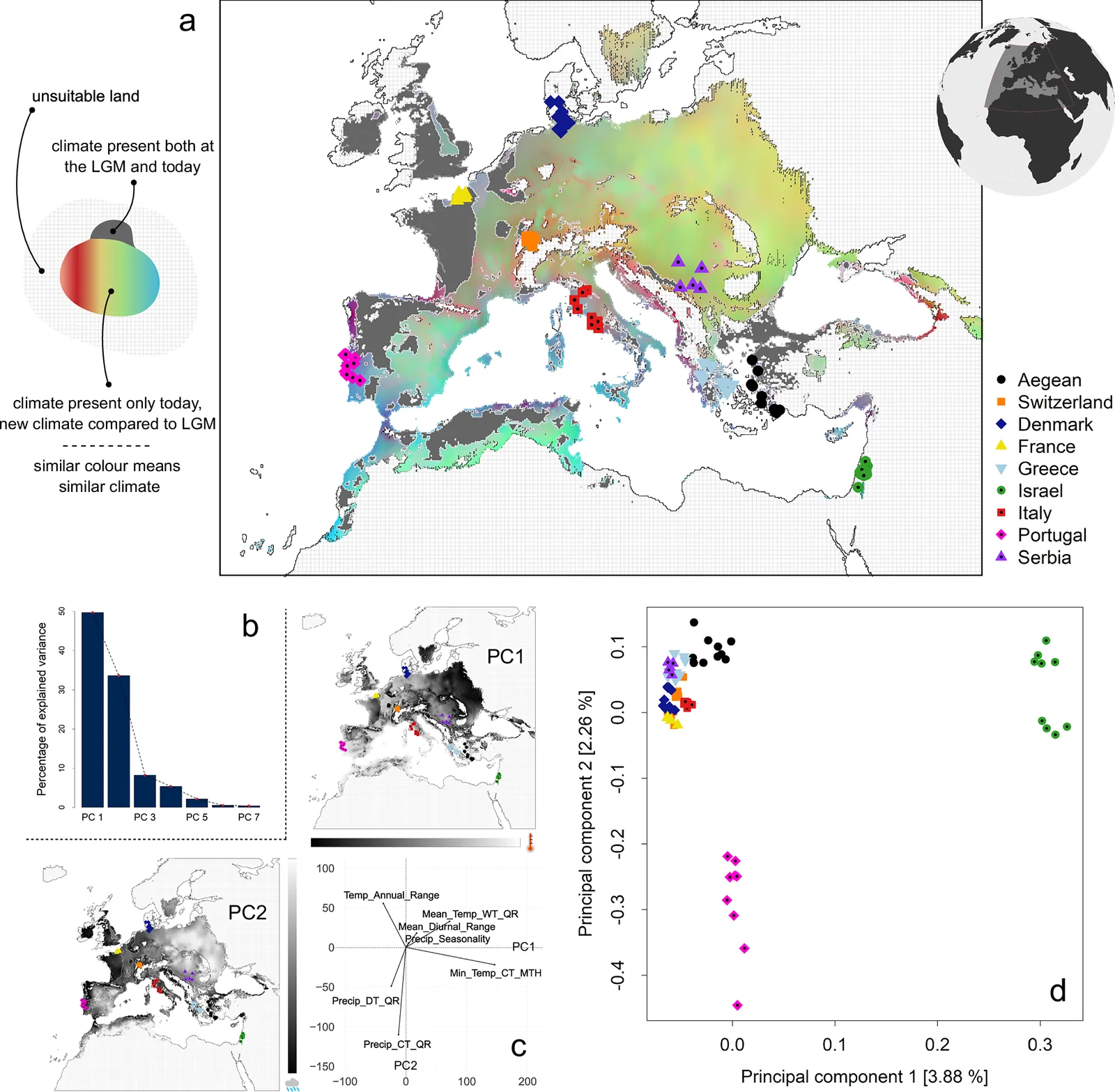
Environmental and genetic variation across the suitable range of the European barn owl. **a** Map depicting the climatic heterogeneity across the range of the European barn owl according to the species distribution modelling. Dark grey cells surrounded by a white border have suitable climatic conditions similar to conditions present during the Last glacial Maximum. Coloured cells outside the white-bordered polygon have climatic conditions not present during the Last Glacial Maximum. Colours are based on the multidimensional climatic space of bioclimatic data shown in (**b**): the scores of the first three principal components (PCs) were converted into values of RGB (PC1: red; PC2: green; PC3: blue) to represent variation in climate. Similar colours represent similar climates. Symbols represent sampling coordinates of individuals from 9 different populations. A jitter has been added for better visualisation (longitude: 0.425; latitude: 0.42). **b** Variance explained by the 7 first principal components of the PCA made on bioclimatic variables from the entire study area pictured on (**a**). Climatic variables came both from the Last Glacial Maximum and today to picture the overall climatic variability. **c** Correlation between climatic variables and the two first axes of the PCA. Dark to white gradients picture the contribution of each axis projected at the European scale - Abbreviations : CT = Coldest ; DT = Driest ; MTH = Month ; Precip = Precipitation ; QR = Quarter ; Temp = Temperature ; WT = Wettest (**d**) PCA based on the whole genome of the 74 European barn owls identified in (**a**). Symbols legend is the same as for panel (**a**). Only the two first principal components are represented.

To identify the climatic factors defining the climatic niche of the barn owl, we performed a Principal Component Analysis (PCA) fitting a multidimensional climatic space both from the Last Glacial Maximum and modern periods. The first three principal components explain 91.50% of the variance (Fig. 1b). The first component, explaining 49.66% of the variance, is driven by temperature-related variables - such as the Mean Temperature of the Coldest Month (bio6), Temperature Annual Range (bio7), and Mean Temperature of the Wettest Quarter (bio8) - which differentiate the Mediterranean region from the rest of Europe (Fig. 1c). The second component, explaining 33.6% variance, is mainly influenced by precipitation factors, including Precipitation Seasonality (bio15), Precipitation of the Driest Quarter (bio17), and Precipitation of the Coldest Quarter (bio19), effectively distinguishing coastal areas from the continental interior (Fig. 1c). The third component, explaining 8.25% variance, is largely driven by the Minimum Temperature of the Coldest Month (bio6; Supplementary Fig. 2 and 3).

We next explored the overlap between past and present climatic conditions in areas suitable for barn owls and showed that owls now occupy a wider variety of climatic conditions than during the Last Glacial Maximum^24^. We found that only a small fraction of today’s suitable habitats shares characteristics with those from the Last Glacial Maximum. We found that only 25.54% of modern suitable cells exhibit Last Glacial Maximum-like climatic conditions (Fig. 1a, dark grey areas). These overlapping regions are found in northern Africa, the northwestern Iberian Peninsula, western France, the British Isles, and western Turkey. In contrast, 74.45% of current suitable cells are characterised by climatic conditions that did not exist during the Last Glacial Maximum (Fig. 1a, RGB-coloured areas). This significant shift suggests that most of today’s barn owl habitats are defined by new climatic conditions, which may have driven local adaptation. We further tested this hypothesis by examining whether barn owls have undergone genomic changes in response to these newly emerged environments.

### Species-wide sampling reveals that a substantial portion of the genome is under climatic selection

To investigate whether the climatic changes since the Last Glacial Maximum have left genomic signatures of local adaptation, we sampled 74 individuals from nine populations across the climatic and European geographical distribution of the species (Supplementary Fig. 4 and 5, Supplementary Data 1). After sequencing of their whole genome, we identified 12,309,943 Single Nucleotide Polymorphisms (SNPs). The overall differentiation was low (overall *F*_ST_ = 0.034) and in line with previous estimates^15^. The first axis of the genomic PCA (explaining 3.88% of the total variance) contrasted individuals from the Levant populations (Israel—IS) to all other individuals (Fig. 1d). The second axis of the PCA (explaining 2.26% of the variance) opposed individuals from the most diverse population (from the Iberic peninsula—PT) to all others (Fig. 1d, Table 1). Overall, southern populations (PT, IT, GR, AE, IS) were more genetically diverse than northern populations (CH, FR, DK, SB) (Table 1). Next, we employed two complementary approaches - a genome scan for selection and a Genotype-Environment Association (GEA) analysis - to pinpoint genomic regions potentially influenced by these climatic conditions.

**Table 1:** Genetic diversity estimated for 9 populations of Western Palearctic barn owls

Population	N	# PolymorphicS	# PrivateA	# RareA	PopSpecificFST
AE	10	4,231,387 (44,618)	193,178 (16,440)	687,423 (28,433)	0.03
CH	9	4,259,867 (19,877)	170,886 (4123)	642,071 (9990)	0.038
DK	10	4,102,932 (17,633)	135,566 (3939)	560,136 (9451)	0.049
FR	4	3,722,772 (-)	124,430 (1292)	487,175 (1859)	0.045
GR	9	4,176,443 (19,842)	144,856 (3756)	606,164 (8453)	0.046
IS	9	4,402,055 (12,195)	626,578 (7595)	1,173,410 (6991)	0.009
IT	9	4,120,120 (10,620)	214,759 (2352)	679,772 (5274)	0.054
PT	9	4,678,275 (25,247)	492,983 (7476)	1,090,608 (12,569)	−0.018
SB	5	4,005,667 (-)	106,496 (2004)	514,405 (2428)	0.06

Standard deviations are found between brackets. See Material and Methods for details on calculation. N, sample size; # PolymorphicS, number of polymorphic sites; # PrivateA, number of private alleles; # RareA, number of rare alleles; PopSpecificFST, population-specific FST computed at the whole genome level.

In our first approach, we scanned the genomes for signatures of selection using population-specific FST^27^. This metric, which accounts for population structure, enables the detection of regions of the genome where individuals have higher genetic similarity than what is observed along the rest of the genome - a pattern possibly resulting from selective events^27,28^. Out of 52,429 windows of 100 kbp (with a 20 kbp slide), we identified 10,607 outlier windows: 7034 were unique to a single population, and 3573 were shared between at least two populations. On average, each population had about 1756 outlier windows (Supplementary Table 1), with Israel and France at the extremes (989 and 2087 outliers, respectively).

To ensure that selection signals were caused by climatic conditions, we directly linked genetic variation to temperature and precipitation conditions with a Genotype-Environment Association (GEA). We conducted a Redundancy Analysis (RDA, Supplementary Fig. 6)^29^ that we integrated into genomic windows with the Weighted-Z Analysis (WZA) method^30^. We detected 2181 outlier windows significantly linked to temperature and precipitation variables (Supplementary Fig. 7) and overlaid it with the population-specific FST scans.

**Fig. 2:**
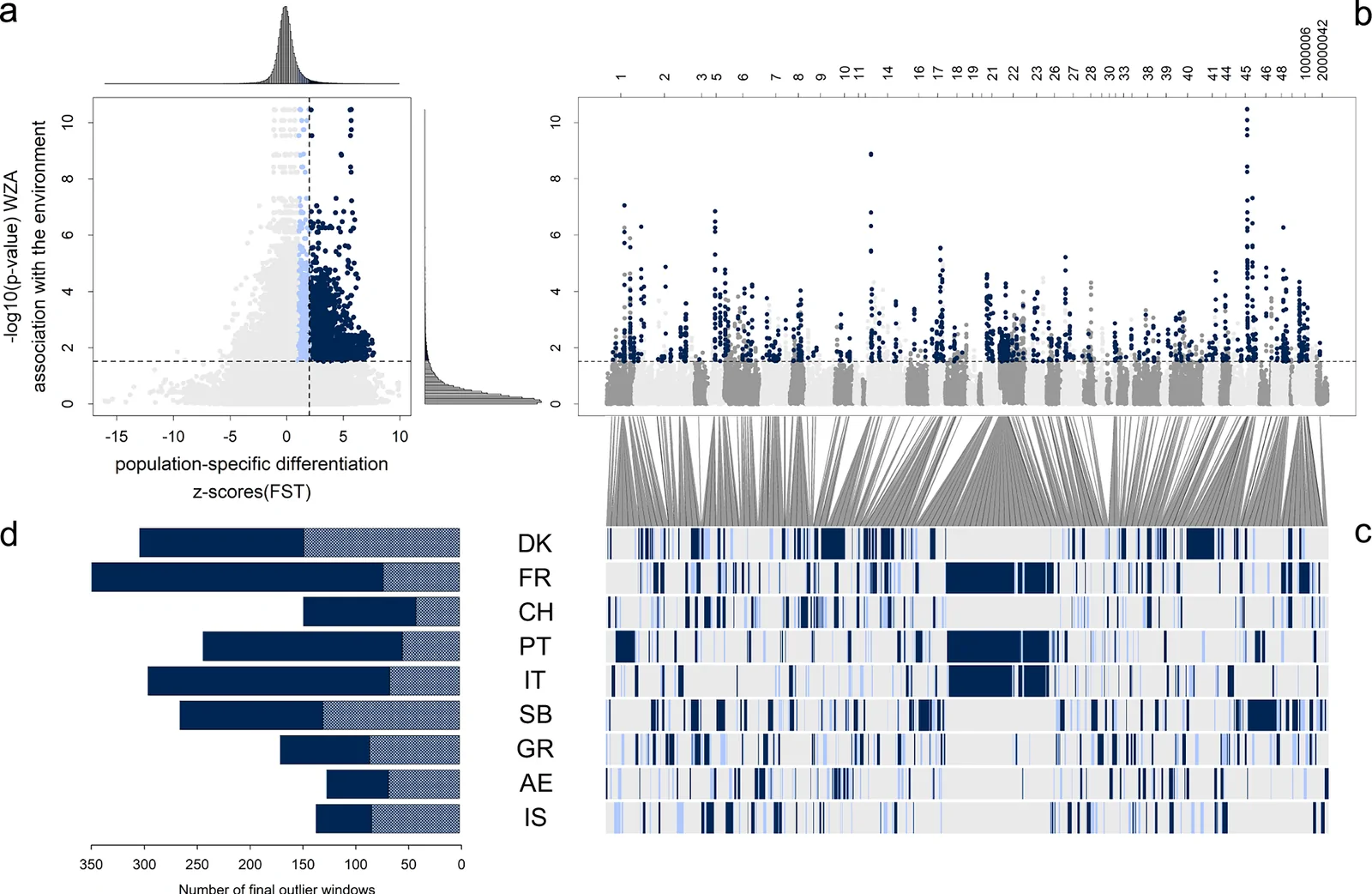
Genomic signatures of selection linked to climate in the European barn owls. **a** Scatterplot of genomic windows (100kbp each) across nine populations. Vertical and horizontal dashed lines are at 2 standard deviation from the mean of z-scores (population-specific FST) and z-scores (*p*-values) of WZA respectively. Each colour presents a class of windows. Dark blue dots represent outlier windows in both population-specific FST and WZA scans. Light blue dots are outlier according to WZA and have a population-specific FST higher than 1 standard deviation from the mean; **b** Genome-wide distribution of the WZA score. Dark blue dots corresponding to the windows identified in panel (**a**). A switch between light and dark grey represents a change in the scaffold. The names of all scaffolds are displayed on the upper x-axis. **c** Distribution of outlier windows in the different populations. Each row represents a population (DK: Denmark; FR: France; CH: Switzerland; PT: Portugal; IT: Italy; SB: Serbia; GR: Greece; AE: Aegean islands; IS: Israel). Each column is a window along the genome, coloured according to the classification in (**a**). **d** Barplot of outlier windows per population. Dark blue bars picture the outlier windows shared with at least one other population while white dashed bars picture the number of outlier windows unique to each population.

This combined analysis yielded a refined list of 1246 outlier windows (displayed in dark blue in Fig. 2a; Supplementary Table 1). 59.71% were unique to a single population while the rest was shared between at least two populations. In these windows, 13,703 SNPs were significantly associated with the climatic variable by the RDA analysis (reported in Supplementary Data 2). By merging successive outlier windows, we delineated 270 genomic regions, many forming distinct adaptive peaks along the genome. Among these 270 regions, 159 presented signs of ongoing selective sweep (Supplementary Figs. 8 and 9). Particularly striking associations were observed on Super-Scaffold 14 and 45 (Fig. 2b). Additionally, we detected a high density of outlier windows in the first half of Super-Scaffold 22 (Fig. 2b-d); this signal, shared by owls from France, Italy, and Portugal, corresponded with a marked increase of population-specific FST in these genomic regions (Supplementary Fig. 10), indicating that individuals within each of these populations are significantly more similar to one another than to the rest of the samples.

To explore the functional implications of selection and climatic adaptation, we extracted a total of 550 genes from the 270 genomic regions that showed signatures of selection and variants associated with climatic variables. Among them, 324 genes were unique to a single population (Supplementary Table 2). Within the 550 extracted genes, Gene Ontology Enrichment (GOE) analysis produced no significant terms with the chicken reference (Supplementary Data 3). Using the human reference yielded a single significant term, namely pancreatic secretion (Supplementary Data 4).

### Differential selection between Southern and Northern populations is driven by climatic differences

**Fig. 3:**
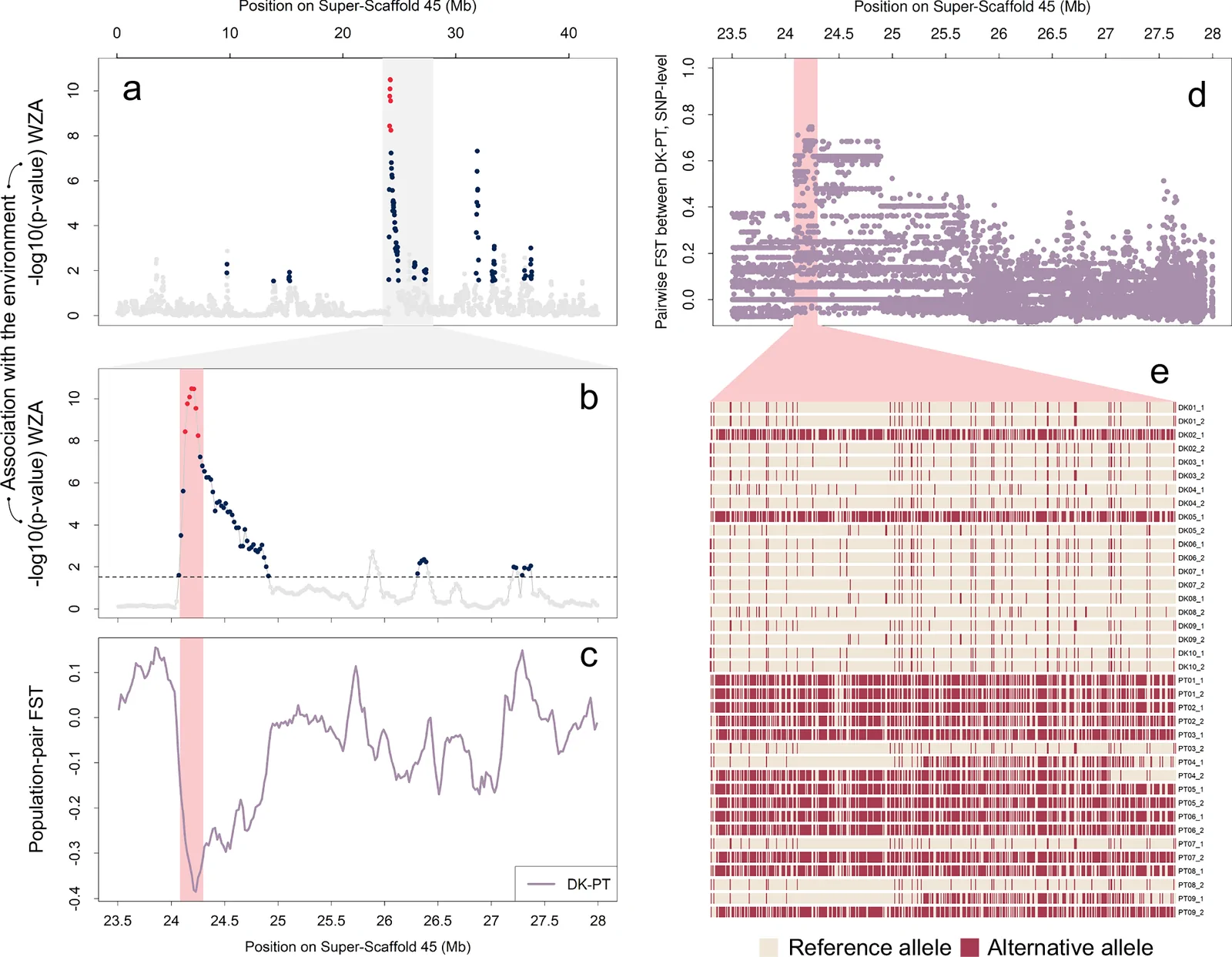
Divergent selection drives the strong climate-driven signal on Super-Scaffold 45. **a** WZA signal across Super-Scaffold 45 with the main peak of outliers highlighted in grey. Dark blue circles are outlier windows from WZA and population-specific FST. Red circles represent outliers with a -log10(*p*-values) higher than 8. **b** Zoom on this peak of the Super-Scaffold 45. **c** Population-pair FST in this region between two populations at the extreme of the first climatic axis in Fig. 1b (namely Denmark and Portugal). Lower value indicates a higher divergence in this region compared to the rest of the genome. **d** Pairwise FST between Denmark and Portugal, computed on a SNP-basis. **e** Genotypes of each SNP within the highlighted region (red rectangle on panel (B), (C) and (D)), using phased haplotypes from Denmark and Portugal. Beige represents the reference allele, and red represents the alternative allele.

Building on these genome-wide patterns, we next focused on specific regions showing strong climatic associations and population differentiation. Our analysis of the region with the highest signal of association with climate reveals that differential selection has led to marked genetic differentiation between populations at opposite ends of the temperature gradient. To explore the signal located on Super-Scaffold 45, we first examined climatic association values within this genomic region (Fig. 3). Out of the 8 outlier windows showing the highest association with climate (red dots in Fig. 3a-b), all were outliers in the population-specific FST scan for the Danish population, and two of them were also outliers in the Portuguese population, two populations at the opposite ends of the temperature gradient (first axis of Supplementary Fig. 5). We assessed the extent of genetic similarity in this genomic region between the two populations by computing the population-pair FST, an estimate of the standardised mean kinship of individuals^31^. With this statistic, we detected a substantial reduction of the genetic similarity between the individuals from these two populations within the region harbouring the highest association with climatic conditions (red rectangle on Fig. 3c). Consistently, we observed an increase of genetic dissimilarity by using a pairwise FST computed on an SNP basis between the two populations (Fig. 3d). We examined the haplotypes within the highest section of the peak which showed a clear distinction between the Danish and Portuguese populations (Fig. 3e).

### A large haploblock is linked to climatic adaptation in past refugia

**Fig. 4:**
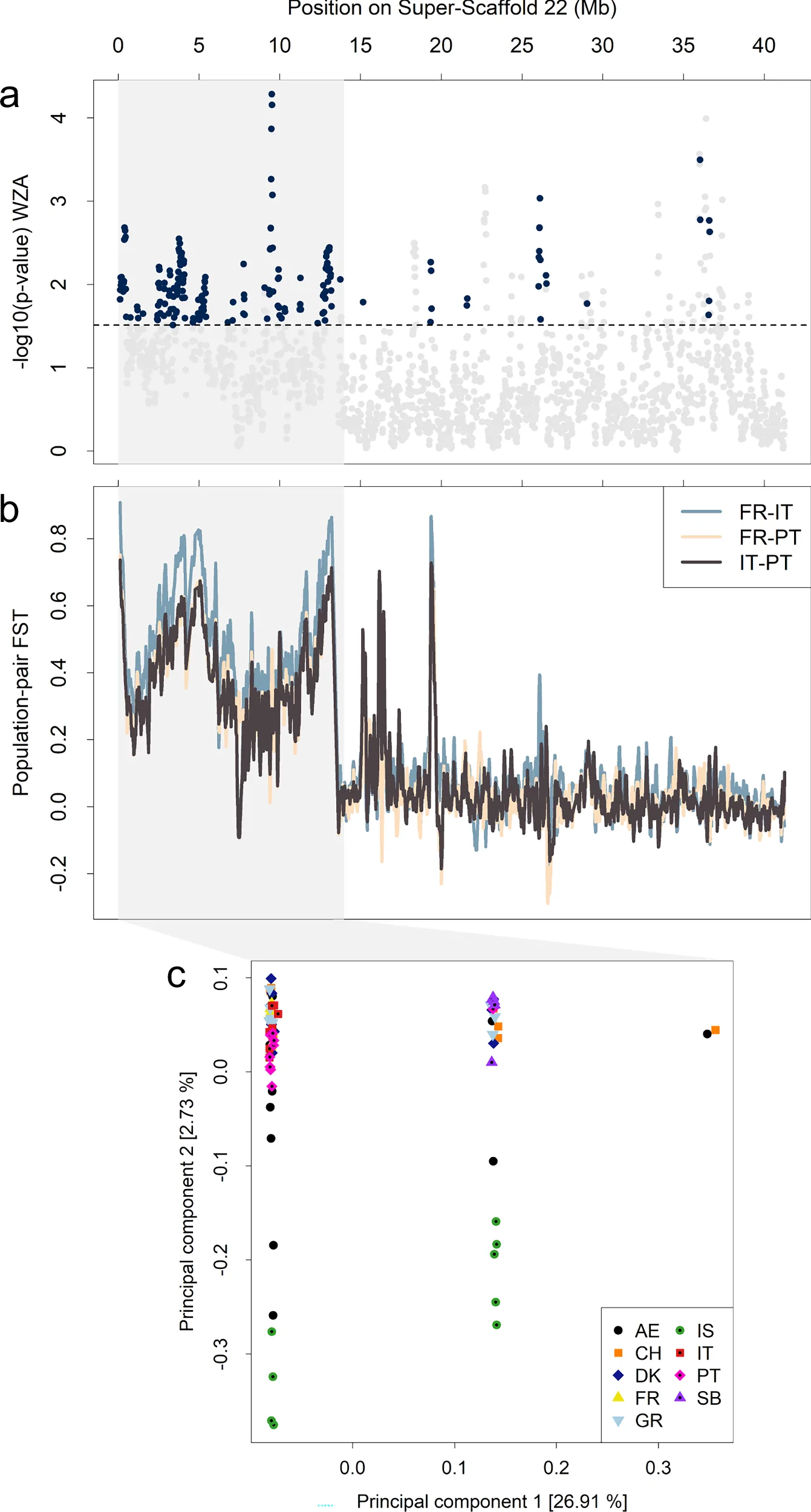
The shared signal in southern populations point to a haploblock linked with local adaptation in the European barn owl. **a** WZA signal along Super-Scaffold 22 with the first 14 Mb highlighted in grey. Dark blue circles are outlier windows from WZA and population-specific FST. **b** Population-pair FST along the entire Super-Scaffold 22, higher value indicates a higher similarity in this region compared to the rest of the genome. As in (A), the first 14 Mb are highlighted in grey. Pairwise comparisons include France (FR), Italy (IT) and Portugal (PT). **c** PCA made on the first 14 Mb of the Super-Scaffold 22, (120,953 SNPs - DK: Denmark; FR: France; CH: Switzerland; PT: Portugal; IT: Italy; SB: Serbia; GR: Greece; AG: Aegean islands; IS: Israel).

Extending our analysis to other regions of interest, we identified a long consecutive signal of climatic association shared between populations from France, Italy, and Portugal in the first half of Super-Scaffold 22 (Fig. 2b-c; Fig. 4a). We used the population-pair FST to assess whether the same genomic variants were shared in the three populations. We detected a higher genetic similarity between the three pairs of populations (FR-IT, FR-PT, and IT-PT) within a 14 Mb region at the beginning of the Scaffold than along the rest of the genome (Fig. 4b). This coincides with a climatic convergence in former glacial refugia, where shifting conditions may have contributed to the shared adaptive signals (Supplementary Fig. 11, 12, 13 and 14).

To further investigate the genomic features of this region, we applied a PCA to the first 14 Mb of the scaffold, encompassing 120,953 SNPs, using data from all 74 individuals (Fig. 4c illustrates the first two axes, explaining respectively 26.91 and 2.73% of the variation). Individuals were divided along the first axis into three distinct haploblocks^32^. The leftmost cluster included more individuals than the one on the rightmost part of the x-axis. In comparison, the PCA conducted on the whole genome only separates the Portuguese samples from the rest along the x-axis and the Israel individuals from the rest along the y-axis (Fig. 1d). Consistent with this clustering, linkage disequilibrium (R^2^) among variants in this region was particularly high in heterozygous individuals (i.e., middle cluster), while it was comparatively lower when computed across all individuals (Supplementary Fig. 15), reinforcing the interpretation of reduced recombination within these haploblocks.

## Discussion

Climatic conditions and their past fluctuations are known to have shaped today’s distribution and genetic diversity of many species^1–3^. While genetic local adaptation is widely recognised as a key mechanism driving species persistence^6^, its role in shaping populations’ capacity to respond to future climatic shifts remains unclear. In this study, we integrated niche-based species distribution modelling (SDM^22^) with genome-wide analyses to investigate how climate-driven changes since the Last Glacial Maximum have shaped the genetic composition and adaptive potential of the Western Palearctic barn owl. First, we showed that suitable conditions nowadays are more climatically diverse than the ones from the Last Glacial Maximum^24–26^, exposing the individuals to heterogeneous and new selective pressures. Then, we identified genomic regions harbouring strong signals of selection and carrying variants associated with climatic variables. We observed signals of genomic adaptation in all the population we sampled across the species’ range, with a particularly strong signal shared among southern populations. Overall, our findings challenge the hypothesis that local adaptation primarily occurs at recolonising margins^33,34^, showing instead that genetically diverse populations such as past glacial refugia are a key source for adaptation^35^. We highlight that refugial populations, by maintaining standing genetic variation, serve as crucial reservoirs of adaptive potential, enhancing species’ ability to respond to future climatic changes^35–37^.

Species distribution modelling of the European barn owl based on its realised climatic niche suggests that the latter is broader nowadays than it was during the Last Glacial Maximum. We used the Maximum Entropy method^38^ for fitting the SDM and project it onto past climatic conditions to compare the extent of suitable habitat under current versus historical climates. Our results indicate that some contemporary climatic conditions along the Atlantic coast, up to the UK, were already present 20,000 years ago. This supports the hypothesis that, as temperatures rose at the end of the Last Glacial Maximum, favourable conditions shifted toward northwestern Europe, facilitating barn owl range expansion^39^. However, we also identified newly emerged suitable climatic conditions that were absent during the Last Glacial Maximum in recolonised Central and Eastern Europe as well as in the glacial refugia where conditions changed for resident populations. All these results suggest that the realised climatic niche of the barn owl was contracted during the Last Glacial Maximum and extended with the subsequent warming. We advance that genetic adaptation played a significant role in this process and further explored this hypothesis.

By combining population-specific F_ST_ scans^27,28^ to identify genomic regions under selection with genome-environment association approaches, we found genomic evidence of local adaptation to climatic variables (humidity and temperature) in all populations. We acknowledge that our use of relatively large genomic windows (100 kbp) may lead to false‑positive associations. Large windows increase statistical power and improve robustness against noisy SNP-level variation (Supplementary Fig. S16), but they can also merge distinct signals or tag broad regions with heterogeneous LD patterns, potentially inflating the number of climate-associated windows^40^. Our integrative approach (combining population-specific FST with WZA and requiring overlap between methods, further confirmed by the selective sweep analyses) substantially reduces this risk, but does not eliminate it. Nonetheless, across the species’ range, we detected 550 genes located within regions significantly associated with environmental conditions and under selection. Among the 550 genes identified in these regions, no significant enrichment for specific pathways was found. We note, however, that GO analyses may be suboptimal, as GO terms composed predominantly of large genes are more likely to be tagged by genomic selection signals than terms containing smaller genes^41^, and further work will be needed to identify the specific pathways and genes involved in the local adaptation of populations. Thus, despite the lack of significant pathway enrichment, the extent of the genome impacted by the climate reinforces the view that local adaptation is a complex, polygenic process involving numerous loci distributed across the genome. Our approach, which integrates differentiation-based and environment-association methods, is recommended to detect signals of adaptation in natural populations and provides complementary evidence for selection^42^. A closer look at the regions of the genome with many variants linked to the environment and with strong traces of selection revealed a wide range of mechanisms shaping the barn owl’s local adaptation, from opposite directional selection to recurrent evolution.

The region located on the Super-Scaffold 45 showed a clear signal of association with climatic variables. In this region, population-specific FST highlights the high differentiation between the Portuguese and Danish populations, which lie at the opposite ends of the temperature gradient (Supplementary Fig. 5). This, along with their distinct haplotypic structure (Fig. 3), suggests that different alleles or haplotypes are under selection in these populations^43^. A closer look at the genes present in the region did not reveal any genes that can easily be linked to temperature adaptation and further work should confirm whether this region plays a role in thermal adaptation.

Our results also revealed a strong and consistent genomic signal of selection linked to climate across all individuals from three populations, one in a region with a climate similar to that of the Last Glacial Maximum (France) and two in past glacial refugia (the Iberic and Italian peninsulas). The size of the region involved, its pronounced pattern of genetic differentiation compared to the rest of the populations as well as the cluster patterns found by our local PCA indicate the presence of an haploblock in this region^32^. Such haploblocks, which often exhibit reduced recombination, have been increasingly linked to local adaptation in the literature^44–46^. Therefore, we suggest that this haploblock may play an adaptative role under specific climatic conditions, but further work is needed to confirm this hypothesis.

The history of this haploblock remains also to be clarified. The pattern of selection is shared between the two peninsulas that hosted the species during the Last Glacial Maximum. Considering the isolation of the two populations at the time and the reduced connectivity nowadays (see Fig. 4 in Cumer et al., 2021 for details)^27^, we suppose that this haploblock predates the Last Glacial Maximum, and that independent and recurrent selection drove the increase of its frequency independently in these two populations. The presence of the haploblock in other populations is most likely due to subsequent gene flow after the Last Glacial Maximum, although additional tests are needed to confirm this scenario.

A key question when studying local adaptation is the origin of adaptive alleles, either arising de novo or from standing genetic variation. Given the pattern of genomic signals we observed, standing variation is likely to have played a central role in this process^47,48^. While de novo mutations can also contribute to adaptation by introducing new beneficial alleles^47,49^, their impact is limited by low mutation rate^50,51^ and the even lower probability of such mutations being both advantageous and occurring in the right environmental context^52^. The reasoning is consistent with theoretical and empirical studies^42,48^, which emphasise that standing genetic variation is often the primary substrate for local adaptation, especially under spatially heterogeneous selection. This whole process has been reported in species as diverse as insects (*Drosophila melanogaster*^53^), birds (Darwin’s finches^54^) mammals (humans^52,55^) and fish (sticklebacks^56^). We did not explicitly test whether local adaptation in barn owls resulted from de novo mutations, allele sorting from standing variation, or a combination of both. Therefore, additional work, focusing on the date of mutations^57^ as well as the timing of selection^58^, should confirm our results. However, the pattern of local adaptation that we identified in populations from past glacial refugia already provides insight into how standing variation fuels adaptation during climatic fluctuations.

The climatic variation since the Last Glacial Maximum provides a valuable framework to gain knowledge on where and how populations adapt to climate change. A wide body of literature explored many aspects of what happens in populations that expanded into newly available habitat, from dispersal limitation^59,60^ to the genetic load accumulated during an expansion^61–65^, and how these mechanisms interact with local adaptation^66–69^. However, the fate of refugial populations remains, to our knowledge, understudied. In this work, we first highlight that the climatic conditions experienced by these populations have changed over time, creating ongoing pressures for adaptation. This suggests that adaptation in refugial populations is not static but a recurrent and dynamic process, a pattern likely shared across many taxa that persisted in historical refugia. Based on our results, we propose that adaptation in refugial populations is a recurrent and dynamic process. As the climate continues to shift, species’ optimal niche moves northward, and southern populations will be among the first to experience novel climatic conditions. These populations often harbour the highest levels of genetic diversity^17^, which likely confers them the greatest adaptive potential in the face of climate change. This underscores the importance of conserving these genetically diverse populations, whose adaptive capacity may be critical for the long-term resilience of the species. While we acknowledge that the pace of current climate change is far more rapid compared to shifts since the Last Glacial Maximum, our study lays the groundwork for a deeper understanding of the genomic foundations of adaptative potential, a key component of resilience across taxa. Future research will be essential to uncover the mechanisms that enable diverse species to persist, adapt, and thrive amid accelerating environmental change.

## Material and methods

### Ecological modelling

#### Species distribution modelling

We first conducted species distribution modelling (SDM^22^) to identify suitable areas for the species during the Last Glacial Maximum and nowadays in the Western Palearctic. Although the species also occurs in other regions such as sub-Saharan Africa, we restricted our modelling to the Western Palearctic because previous phylogeographic studies indicate that present‑day European populations mainly recolonised the continent from Iberian and Italian refugia^15^. Extending the modelling further south would therefore introduce regions that were unlikely to contribute to the genetic ancestry of the populations studied. We fitted SDM using the Maximum Entropy modelling software (MaxEnt^38,70^, v.3.4.3), a presence-only based procedure in a similar approach as the one described in Cumer et al. (2022)^15^.

First, we extracted 19 bioclimatic variables at a 5 arc-minute (~9.3 km at the equator) resolution from the WorldClim 1.4 database^71^ using the rbioclim R package^72^. To avoid redundancy between variables, we removed variables with a correlation equal to or higher than 0.8^73^, leading to a set of 7 uncorrelated climatic variables: Mean Diurnal Range (Bio2), Min Temperature of Coldest Month (Bio6), Temperature Annual Range (Bio7), Mean Temperature of Wettest Quarter (Bio8), Precipitation Seasonality (Bio15), Precipitation of Driest Quarter (Bio17), and Precipitation of Coldest Quarter (Bio19).

We performed the analysis using the dismo R package^74^ v.1.3-14 on R v.4.3.2. To determine which combination of parameters optimised the model without over-complexifying it^75^, we built models with linear, quadratic, and hinge features, using a range of regularisation multipliers from 1 to 5 (as recommended in Warren & Seifert, 2011^76^). A quadratic feature with a regularisation multiplier of 1 yielded the lowest AIC and was chosen for further modelling. We ran a total of 100 quadratic MaxEnt models (with a regularisation multiplier = 1), omitting 25% of the data during training to test the model and using 10’000 background points randomly sampled across the study area. For each model, we randomly sampled 1000 points within the species’ geographic range as defined by the IUCN distribution map^77^, rather than using actual occurrence records. These points represent potential presence locations within the known range and were generated using the spsample() function implemented in the sp R package^78^, after cropping the ICUN shapefile to Europe and North Africa. We evaluated the predictive performances of the models by assessing the area under the curve (AUC) of the receiver operating characteristic (ROC) plot of the test data^79^. All models had an AUC between 0.756 and 0.807 (see Supplementary Fig. 17), thus classified as fair to good according to Li et al. (2020)^80^.

We projected the 100 models to the present climatic conditions and to the climatic conditions from the Last Glacial Maximum (about 20’000 years ago), also extracted from the WorldClim 1.4 database (scenario CCSM from PMIP2^71^) at 5 arc-minute resolution. We used the “maximum training sensitivity plus specificity” (MaxSSS) threshold, as recommended for presence-only data^81^, to transform the projected output from the models into binary suitability maps (0 unsuitable, 1 suitable). Finally, we averaged the values among replicates and retained cells as suitable only if they were so in at least 90% of the models. To avoid model extrapolation when projecting the models in the past, we used the Multivariate Environmental Similarity Surface (MESS) approach^82^ to identify and discard areas from the past with climatic conditions absent from those in the calibration data.

#### Identification of newly suitable conditions absent from the Last Glacial Maximum

Then, we determined whether currently suitable areas display climatic conditions different from those found during the Last Glacial Maximum. To do so, we extracted values of the 7 climatic variables used in the SDM (see Species distribution modelling section for details) at every continental cell of the studied area for both past and present and performed a Principal Component Analysis (PCA) to represent the environmental space. We retained the first three principal components that explained up to 91.50% of the climatic variance. In this environmental space, we generated a “multidimensional climatic space”, representing the entire range of climatic conditions suitable for the barn owl, by combining past and present suitable pixels within this PCA space. We then characterised the past suitable conditions for the species by creating a polygon around the Last Glacial Maximum suitable conditions, using the alphashape3d R package (α = 0.5, keeping all the data^83^). Next, we classified the suitable conditions at current time as located inside or outside the polygon, thus respectively present or absent from the range of Last Glacial Maximum suitable conditions.

#### Estimation of the shift of climate within suitable areas

To quantify shifts in climatic conditions between Last Glacial Maximum and now, we identified cells that were suitable both during the Last Glacial Maximum and present time (Supplementary Fig. 11) and computed the Euclidean distance between their past and present positions in the multidimensional climatic space. We then rescaled the distance values from 0 to 1 (Supplementary Fig. 12).

Because we discovered a strong signal of selection shared between the two glacial refugia populations (see Results section), we investigated how the climate has changed since the Last Glacial Maximum in the two peninsulas to assess whether the climate converged or diverged in these two locations. We extracted values of the 7 climatic variables used in the ecological modelling for the Last Glacial Maximum and today (see Species distribution modelling section) at the GPS coordinates for the Italian and Portuguese samples. We projected these conditions inside the multidimensional climatic space (Supplementary Fig. 13). Then, we computed the pairwise Euclidean distances between all sampling localities of the two peninsulas during the Last Glacial Maximum and the present time (8 (IT) x 8 (PT) pairwise distances at both time points). This way, we quantified the climatic difference between the Italian and Portuguese sampling localities at the two different time points (Supplementary Fig. 14).

### Individual barn owl sampling

#### Biological samples

This study took advantage of the datasets of European barn owls previously published by Cumer et al. (2022), Machado, Cumer, et al. (2021), and Machado, Topaloudis, et al. (2021)^15,27,84^. We retrieved the whole genome sequences from the Sequence Read Archive (SRA - Bioprojects PRJNA700797, PRJNA727915, and PRJNA727977, Supplementary Data 1).

#### Genetic data preparation

The bioinformatics pipeline used to obtain analysis-ready SNPs was adapted from the Genome Analysis Toolkit (GATK) Best Practices^85^ to a non-model organism following the developers’ instructions, and adapted from Cumer et al. (2022)^15^. Raw reads were trimmed with Trimommatic v.0.36^86^ and aligned to the reference barn owl genome^39^ with BWA-MEM v.0.7.15^87^. Base quality score recalibration (BQSR) was performed using high-confidence calls obtained from two independent callers – GATK’s HaplotypeCaller and GenotypeGVCF v.4.1.3 and ANGSD v.0.921^88^ – as a set of “true variants” in GATK v.4.1.3 following the procedure described in Machado et al. (2021)^39^.

Genotype calls were filtered for analyses using a hard-filtering approach as proposed for non-model organisms, using GATK and VCFtools^89^. Calls were removed if they presented: low individual quality per depth (QD < 5), extreme coverage (1100 > DP > 2500), mapping quality (MQ < 40 and MQ > 70), extreme hetero or homozygosity (ExcessHet > 20 and InbreedingCoeff > 0.9) and high read strand bias (FS > 60 and SOR > 3). Then, we removed calls for which up to 5% of genotypes had low quality (GQ < 20) and extreme coverage (GenDP < 10 and GenDP > 40). We kept only bi-allelic sites, excluded SNPs on the sexual chromosome (Super scaffolds 13 and 42^39^) and an exact Hardy-Weinberg test was used to remove sites that significantly departed (*p* = 0.05) from the expected equilibrium using the R package Hardy Weinberg^90,91^.

To prevent some alleles from being over-represented in the dataset (relatedness statistic based on the method of Manichaikul et al., 2010^92^, implemented in VCFtools v0.1.14^89^), we identified pairs of individuals with a relatedness higher than 0.1. We removed one of the two individuals for each identified pair, leading to a dataset of 74 individuals (Supplementary Data 1).

We then excluded genomic regions with uncertain SNP calling by removing regions of the genome where our ability to confidently map reads is limited (i.e., a “mappability” mask). To achieve this, we followed the procedure documented at lh3lh3.users.sourceforge.net/snpable.shtml. In summary, we divided the reference genome into reads of 150 base pairs (bp) with a sliding window of 1 bp. These artificial reads were then mapped back to the reference using BWA-MEM v.0.7.17. Regions of the sequence where less than 90% of the reads mapped perfectly and uniquely were discarded by excluding variants using a bed file in VCFtools v0.1.14. We retained only bi-allelic SNPs, removed loci with more than 5% missing data, and excluded from the analysis 15 scaffolds (out of 60) showing less than 1000 SNPs and one extra sexual scaffold.

At the end of the filtration process, we retained a set of 12,309,943 SNPs from 39 scaffolds, genotyped in 74 European barn owls (10 individuals from the Aegean Islands (AE), 10 from Denmark (DK), 4 from France (FR), 9 from Greece (GR), 9 from Italy (IT), 10 from Israel (IS), 9 from Portugal (PT), 5 from Serbia (SB), and 9 from Switzerland (CH)).

To explore the genome-wide variations of the Western palearctic populations and check its concordance with previous results, we conducted a PCA with the SNPRelate R package^93^ using this entire set of SNPs and individuals. Additionally, we assessed genetic diversity among the 9 sampled populations following the procedure of Cumer et al., (2022)^15^. Briefly, we identified the number of polymorphic sites, private alleles, rare alleles and the whole genome population-specific FST for each population independently. To account for differences in sample sizes (ranging from 4 to 10), we randomly sampled 5 individuals from each population - except for FR and SB - and calculated these diversity estimates on the resulting subsets. This resampling process was repeated 10 times, and we reported the mean and standard deviation of the diversity estimates.

#### Phasing process and evaluation

We performed the phasing and imputation of the individuals’ genotypes in two steps. First, we conducted a read-based phasing of each individual using WhatsHap v1.0^94^. During this step, we reconstructed haplotypes based on the mapped sequencing reads covering multiple variants. Between the two filtering steps, we applied a Minor Allele Frequency (MAF) filter to ensure it was higher than 5% using VCFtools v.0.1.14^89^, thereby removing rare alleles from the dataset that could influence the second round of phasing, resulting in a dataset of 4’689’284 SNPs. Then, we conducted the complementary round of phasing with ShapeIt4 v4.1.3^95^ with default parameters. The latter uses a statistical approach to infer individuals’ haplotypes based on the population genotypes^96^ and incorporates the phase information from the read based phasing.

To evaluate phasing performance, we calculated the switch error rate (SER) of the phasing generated by ShapeIt4 for each individual^97^. For each individual, we conducted a statistical phasing using ShapeIt without considering the read-based phasing from WhatsHap for the focal individual. Subsequently, we compared this phasing to the “true” local phasing, inferred from the read-based approach (WhatsHap). We estimated the switch error rate between both sets of phasing using the switchError code (available at https://github.com/SPG-group/switchError). Among the 74 phased individuals, the mean error rate was 2.23 * 10^-4^ and none exceeded 0.7% (Supplementary Fig. 18).

### Detection of traces of selection in each population

To identify genomic regions potentially under selection, we computed the window-based population-specific FST using the hierfstat R package^98^. The statistic was calculated across the genome in overlapping windows of 100 kbp with 20 kbp steps for each independent population. This 100kbp window size was previously used in the species for similar analyses^27^. Only windows containing at least 250 SNPs, corresponding to two standard deviations below the mean, were included in the analysis.

To identify outlier windows (regions with extreme values of positive population-specific FST), we first transformed the statistic into Z-scores by subtracting the population mean from each estimate and dividing it by the standard deviation for each population independently. We then combined Z-scores from all populations and considered a window as an outlier in each population if its Z-score for population-specific FST was equal to or higher than 2 standard deviations from the mean of the merged Z-scores. This approach allowed us to focus on regions exhibiting an excess similarity in the population compared to the rest of the genome (high population-specific FST). For further details about this method, refer to Cumer et al. (2022)^27^. We computed the statistic on the set of 12,309,943 SNPs, unfiltered for Minor Allele Frequency (MAF). To ensure that rare alleles did not influence population-specific FST, we also calculated this statistic on the filtered variants from MAF (set of 12’309’943 SNPs). The high consistency between the two estimates (with and without MAF filtering), as depicted in Supplementary Fig. 19, supported our decision to retain the statistic computed on the unfiltered variants set (12,309,943 SNPs).

To further confirm the signal identified by the population-specific FST approach, we used the Likelihood-based Detection of Selective Sweeps implemented in SweeD^99^, on a window basis. Sweed was run for each population independently as well as across all populations. SweeD grid size parameter (-grid) was adjusted based on chromosome length to obtain a ~10kbp resolution along the genome and using folded mode (-folded) since variants were not polarised. We considered a region as an outlier in each population and across populations if its likelihood was equal to or higher than 2 standard deviations from the mean.

### Genotype-Environment Association

#### Redundancy Analysis

We independently conducted a SNP-basis Genotype-Environment Association (GEA) analysis to assess the relationship between the genotypes of the barn owl and their surrounding environment for all populations simultaneously.

Based on the GPS coordinates of the 74 samples (Supplementary Data 1), we extracted values for the same 7 climatic variables (Mean Diurnal Range (Bio2), Min Temperature of Coldest Month (Bio6), Temperature Annual Range (Bio7), Mean Temperature of Wettest Quarter (Bio8), Precipitation Seasonality (Bio15), Precipitation of Driest Quarter (Bio17), and Precipitation of Coldest Quarter (Bio19)) as those used in the species distribution modelling (see Species distribution modelling section for details). We conducted a Principal Component Analysis (PCA) on these climatic data to assess the level of climatic dissimilarity experienced by the barn owls sampled in this study nowadays. We retained the first three principal components, explaining 94.97% of the climatic variance.

We then associated variants with genomic information using Redundancy Analysis (RDA) with the vegan R package^29,100^. This method relies on a multiple linear regression of the observed genotypes on a set of abiotic or biotic predictors. The expected genotypes based on the model (also called fitted values) are then extracted and used as input for a PCA called RDA space. The projection of the principal axes and components in this RDA space allows the detection of the SNPs that contribute the most to the RDA axis and whose allelic frequency might be putatively driven by the explanatory variables^29^. Since RDA doesn’t support missing data, we used the imputed genotype matrix (phased set of 4’689’284 SNPs, see Phasing process and evaluation section for details) as the response matrix and the bioclimatic variables extracted at each sampling locality for the multiple linear regression.

To evaluate the significance of the relationship between genotypes and climatic variables, we performed a permutation test^101^. In brief, we computed a test statistic (F-statistic) from the regression using the true data. Afterwards, we carried out 999 additional regressions on permuted rows of the response data (i.e., the genotype matrix), allowing us to establish the empirical null distribution of the statistics, to which we compared the observed statistic^101^. From this test, we found a significant relationship between the environmental variables and genetic components (Supplementary Table 3). To select the number of RDA axes to retain, we also performed a permutation test for each axis (*n* = 100) by following the procedure given by Borcard et al. (2011)^101^. Applying this method, we kept the first five RDA axes, explaining 80.41% of the constrained variance (Supplementary Table 4). To detect loci that strongly contribute to the individual’s discrimination in the RDA space (outlier loci), we followed the procedure described in Capblancq et al. (2018)^102^: we computed Mahalanobis distances between each locus and the centre of the RDA space by using the previously retained five axes. *P*-values were adjusted for the false discovery rate (FDR) by computing q-values using the qvalue R package^103^. We considered a SNP as an outlier if its q-value was less than 0.1, following Capblancq et al. (2018)^102^.

#### Weighted-Z analysis

To improve the robustness of our GEA approach, we decided to transform the SNPs *p*-values from the Redundancy Analysis into window-based statistics through the Weighted-Z analysis (WZA) proposed by Booker et al. (2021)^30^. This method takes as input individual p-values from any SNP-based GEA approach and calculates a weighted-Z statistic for a given genomic region. To do so, it transforms the *p*-values of the focal window into z-scores and computes the weighted-Z statistic using the equation provided by Booker et al. (2021)^30^, which considers the variation in the number of SNPs among windows along the genome.

We computed the weighted-Z statistics on the same windows as population-specific FST (size of 100 kbp, step of 20 kbp). This consistency enables direct comparison between the two approaches and aligns with previous empirical studies in this species^27^. While alternative window sizes were considered, we chose the 100 kbp window as a compromise to reduce stochastic noise while retaining the resolution necessary to detect meaningful signals (Supplementary Fig. 16). However, we acknowledge that using large windows may also reduce mapping resolution and increase the risk of false positives by grouping neighbouring but independent associations, a known limitation of window‑based GEA approaches^40^. Since WZA does not support overlapping windows, we split the window set based into five sets of non-overlapping windows. We ran 5 separate weighted-Z analyses, one with each input file, and merged the outputs to obtain the final one. We considered a window as an outlier when the -log10 of its *p*-value was equal to or higher than 2 standard deviations from the mean of all windows (equivalent to a *p*-value of 0.03 assuming a normal distribution).

### Genomic signal of local adaptation to climate

#### Concordance between the genome scans and landscape genomics

Because our study aimed to detect genomic regions potentially shaped local climatic conditions, we defined outlier windows as those overlapping between the genome scans (see Detection of traces of selection in each population section) and the Genotype-Environment Association analysis (see Weighted-Z Analysis section). Such integrative approach that combines GEA with differentiation-based metrics such as FST strengthen inference by reducing false positives and identifying loci under spatially varying selection^42^. We further confirmed that the regions identified harboured signals of ongoing selection by assesing how many of them harboured significant signal of selective sweep (see Detection of traces of selection in each population section). Based on the annotated barn owl genome (GenBank Assembly Accession: GCA_018691265.1), we considered genes that partially or fully overlapped the final list of outlier windows as potentially involved in local adaptation to climate and extracted a list of genes for each population.

#### Gene Ontology Enrichment

We conducted Gene Ontology Enrichment (GOE) analyses using ShinyGo v.0.8^104^ to assess which biological pathways the genes located in the final list of outlier windows could be involved in and whether we could link some to local adaptation to abiotic conditions. We performed one GOE using all genes detected in at least one population. As a baseline set of genes against which we compared the observed enrichment signature, also called the background list, we used all the genes annotated in the barn owl genome that could have been detected with our 52’429 non-overlapping windows from the genome scans or WZA. For each analysis, we used the default pathway databases, namely KEGG (Kyoto Encyclopedia of Genes and Genomes), as well as the GO Biological Process database. GOEs were ran using both the human (*Homo sapiens*) and the chicken (*Gallus gallus*) as reference species.

### Exploration of signals specific to some populations

#### Population-pair FST on Super-Scaffold 45

We identified a strong environmental association in Denmark and Portugal, two populations at the opposite of our environmental gradient, with strong population-specific FST in this region in each of them. To know whether populations have the same genetic variants or opposite ones, we assessed the level of genetic (dis)similarity between pairs of populations on this part of the genome. To do so, we computed a population-pair FST, described in detail in Goudet & Weir (2023)^31^, using the hierfstat R package^98^. In brief, for every pair of populations, we calculated the average kinship among pairs of individuals and standardised it by the average kinship between all populations using the same dataset and windows employed for population-specific FST analysis. A schematic example is provided in Supplementary Fig. 20 for the Denmark - Portugal population pair.

#### Pairwise FST at the SNP level

To calculate a pairwise FST at the SNP level^31^ and confirm the signal obtained through population-pair FST, we used the *fs.dosage* function of the hierfstat R package^98^. We computed this pairwise FST on the entire Super-Scaffold 45 between Denmark and Portugal using the same dataset as for population-specific FST or population-pair FST.

#### Local PCA on Super-Scaffold 22

As we detected a strong signal of selection related to climate in the first half of Super-Scaffold 22, we decided to investigate the genetic architecture of this region. To do so, we conducted a PCA on the first 14 Mb of the scaffold (120,953 SNPs) using all individuals, using the SNPRelate R package^93^.

### Reporting summary

Further information on research design is available in the Nature Portfolio Reporting Summary linked to this article.

## Supplementary information

Transparent Peer Review file

Supplementary information

Description of Additional Supplementary Files

Supplementary Data 1

Supplementary Data 2

Supplementary Data 3

Supplementary Data 4

Reporting Summary

## Data Availability

The data underlying this article are available in the GenBank Sequence Read Archive Database at https://www.ncbi.nlm.nih.gov/sra, and can be accessed within Bioproject PRJNA700797, Bioproject PRJNA727915 and Bioproject PRJNA727977. Additional data can be provided by the corresponding authors.

## Code availability

Code used in this article is available at https://github.com/hugocrvl/TytoAlbaLocalAdaptation.
