# Supplementary material for: Genetically diverse populations hold the keys to climatic adaptation in the Western barn owl (Tyto alba)
Source: Commun Biol. 2026 Jun 3;9:1172. doi: 10.1038/s42003-026-10385-8 (PMC13542081; doi:10.1038/s42003-026-10385-8)
Supplement: Supplementary file 1 [file 42003_2026_10385_MOESM1_ESM.pdf]

nature portfolio

Peer Review File

Genetically diverse populations hold the keys to climatic adaptation in the Western barn owl (Tyto alba)

Corresponding Author: Mr Hugo Corval

This manuscript has been previously submitted at another journal. This document only contains information relating to versions considered at Communications Biology.

This file contains all reviewer reports in order by version, followed by all author rebuttals in order by version.

Version 0:

Reviewer comments:

Reviewer #1

(Remarks to the Author)
This paper provides a valuable contribution on new aspects related to genomic adaptation. I found it good overall and made several suggestions onto the manuscript's PDF. The manuscript's main message is that extant genomic diversity at the core of species' distributions are key to fuel genomic adaptation to climate change, and this is a very relevant point. A very positive aspect of this manuscript is that GEA analyses were performed only in parts of the genome that were found independently to be under selection, a logical approach that sometimes is not practiced in genomic adaptation studies. Overall, the authors do a reasonable good job coinvincing the reader that their data favors the hypothesis that genomic diversity in refugial areas served as the basis for climate adaptation in newly colonized areas. However, I highlighted across the manuscript's PDF several points that need to be addressed by the authors to improve their work's quality, clarity, and consistency. Finally, I suggest exclusing "cosmopolitan raptor" from the paper's title, since the study in focused in the western paleartic, not throughout the world, in fact not taking into account sub-saharian populations of the same species (for more on this refer to my comments onto the PDF).

Reviewer #2

(Remarks to the Author)
Guisan et al. combined species distribution modeling and population genomic approaches to investigate climate adaptation in barn owl. The study documents post-LGM expansion into broader climate niches and identifies putative climate-adaptive variants associated with climate variables. They also detected the climate adaptive variants with a segment of genome inversion. Overall, the study is well designed and sampling size is decent. The species distribution modeling components appear robust, however, several concerns regarding the genomic analyses warrant clarification.

My first concern regards selection detection methodology. The reliance of population-specific FST as the primary selection metric is problematic. FST reflects relative genetic differentiation and is confounded by demographic history, e.g., ancestral polymorphism, population structures and nucleotide diversity. I don’t think FST is an appropriate to detect selection. Furthermore, FST is a pairwise comparison. Authors did not adequately justify the computational approach for estimating population-specific FST across multiple populations. This information is critical for evaluating the results. I would recommend to use regular approaches detecting selective sweep, such as Sweed, number of segregating sites by length (nSL) or the standardized integrated haplotype score (iHS) to detect selective signature.

My second concern regarded genotype-environment association analysis. RDA-based GEA is designed to detect individual SNPs, while the WZA analysis groups linked SNPs. In the submission, 100-kb window size was used for WZA analysis, which appears arbitrary without reference to LD decay patterns (WZA recommendation). This large window size could artificially inflate lineage signals. Furthermore, the requirement for overlap between GEA-detected variants and FST outliers also lacks the theoretical justification. GEA itself is aimed to detect climate-adaptive genetic variants. FST is to estimate relative genetic differentiation. I can’t see the login in this combination.

My third concern referred inversion characterization. The claim of inversion requires strong evidence rather than local PCA

alone. Supplemental analyses should include recombination rate suppression, LD pattern across the putative inversion.

My last concern regarded overstatements in Discussion, in particular regarding interpretations about gene ontology, and climate adaptation of inversion. For example,

Discussion:
"Line 255-272. I think this discussion is too speculative regarding that authors only based a few gene ontology terms. These terms are quite broad and basic functions. For example, in L261 "This finding aligns with previous research showing that pathogens and infectious diseases exert strong selective pressures on both humans and birds40,41"

I don't think you can infer this from your results, with only based a few identified immune related genes. Immune can response many ecological factors, not only pathogens and infectious disease

"Line 263 temperature and rainfall significantly influence pathogen community composition42,4"

Again, temperature and rainfall not only influences pathogen community

"Line 268. This result was consistent with Bergmann's 269 rule which predicts that body sizes of warm-blooded vertebrates negatively correlate with temperature, leading to smaller body sizes in warmer climates45,46 270."

You can't infer a Bergmanns' evidence only based on the gene ontology enriched by genes related developmental process and morphogenesis.

"Line 291-293 Briefly, considering the genomic disruption that breakpoints can cause, as well as the fact that nearly suppressing recombination will prevent purging of deleterious alleles in the inverted haplotype, it is unlikely for a newly appeared inversion to persist and spread in a population51"

I think inferring inversion only by local PCA is not enough. A start-of-art method is to check recombination rate. I did not find recombination estimation in Results, but in Discussion authors referred suppression recombination. Did I miss this in Results? Besides, even the inversion occurred, how it related to climate adaptation?

"Line 303-309 A key question is the origin of adaptive alleles and given the pattern of genomic traces of local adaptation we observed, we propose that standing variation played a crucial role in this process54,55. One possibility for local adaptation to occur is de novo mutations bringing new favourable alleles in the different populations54,56. Considering the low mutation rate57,58 and the even lower probability of a mutation being both advantageous and occurring in the right environmental context59, this mechanism is unlikely to fully explain the extent of genomic regions involved in local adaptation (Figure 2)".

I don't think this is particular true. Regarding the method you used to infer climate adaptation, FST and genotype-environment association, you can only identify standing variation. This sentence seems not necessary as your experimental design was not able to identify de novo mutation.

Methods
Line 419-424. Please provide specific information about your vcf calling and filtering. What is hard-filtering approach? Not every author would go to check Cumer et al. 2022.

Reviewer #3

(Remarks to the Author)
Review of the Article:
Genetically diverse populations hold the keys to climatic adaptation: a lesson from a cosmopolitan raptor

The authors studied local adaptation using the European barn owl, applying Species Distribution Modelling and characterizing climatic conditions in relation to the Last Glacial Maximum. The authors combined a genome scan for selection and a Genotype-Environment Association (GEA) analysis to identify genomic regions and genes potentially involved in the local adaptation of the different populations. I found the methodology particularly well developed and applied for the research question, and I consider that the study would be of great interest to Communications Biology's readers.

I only have 2 main concerns. One is a (1) suggestion to improve the clarity of the results, and the other is a (2) comment in relation to the Gene Ontology Enrichment (GOE) analysis. Some minor comments can be found below, ordered by sections, and specific lines are signaled with L.(#line).
Major comments:
(1) It is somewhat difficult to identify the main message while reading the Results section. The individual parts appear somewhat scattered and disconnected at first glance, and it requires additional effort from the reader to grasp the overarching link. I suggest restructuring the Results and Discussion section with a clearer hierarchical organization, moving from the general (highlighting the main findings) to the specific details. From my perspective, the main point is the genomic signal of selection that the authors found linked to climate, but I see the whole description of the climate as an accessory part

to get there.
(2) The Gene Ontology Enrichment analysis appears to use a very broad approach, which results in outputs that are too general and difficult to interpret. I recommend refining the analysis with a more specialized avian annotation. For example, in ShinyGo v0.8106 the Gallus gallus genome could be selected as a reference, or alternatively, the authors could build a customized annotation tailored to their dataset. It is also not clear if the discussion about the found GO terms in the main text is based on statistically significant results or not, or if they were controlled by multiple testing or not? Finally, I suggest improving the readability of the supplementary material to ensure transparency and reproducibility. For example, the names and headers of the tables can be clearer, the headers of the columns should be meaningful and self-explanatory.

Minor comments:
Title
1. Consider making the title more direct. For example, replacing “Genetically diverse populations hold the keys to” with “Genetic diversity is the key for”. Other options: “Genetic diversity underpins past and future climate adaptation in the barn owl”, or “Genetic diversity as the foundation of barn owl resilience to climate change”, etc.

Abstract and Introduction
2. Please avoid the unnecessary use of the word “demonstrate”, instead, use a softer one like “show” unless there is really strong evidence for a statement.
3. L 66 I suggest choosing one consistent style to mention the Last Glacial Maximum (LGM) across the text from this line. I think keeping the whole words would be preferred, considering the broader audience of this journal.

Results
4. See major comments

Discussion
5. Same as in the Introduction, please replace the word “demonstrated” with a more moderate one.
6. L. 338 I think the article would benefit here from a bit of highlighting the importance of the Barn owl for the European ecosystems. This is something that could also be included in the introduction as part of the importance of the study. I mean, what would be the risk if the Barn owl didn’t have such adaptive capacity?

Materials and Methods
7. Ok, only see my comment about the Gene Ontology Enrichment (GOE) analysis

Version 1:

Reviewer comments:

Reviewer #2

(Remarks to the Author)
This is my second review of this submission. In my initial review, I raised two primary methodological concerns that are central to the validity of the authors' conclusions.

First, the continued reliance on population-specific FST as the primary metric for detecting selection remains problematic. While FST is a useful measure of genetic differentiation, it is strongly confounded by demographic history—including ancestral polymorphism, population structure, and local variation in nucleotide diversity. Consequently, high FST values alone are not robust signatures of selective sweeps. I had recommended supplementing or replacing this approach with established selection detection methods—such as SweeD, nSL, or iHS—which are designed to distinguish selective signals from demographic noise.

Second, the window-based climate association analysis (using 100 kb windows) is susceptible to identifying large false-positive associations, as large windows inevitably contain many SNPs unlinked to the adaptive trait. I previously suggested a more targeted follow-up analysis, such as testing individual climate-associated SNPs identified within these candidate windows, to improve specificity.

Upon re-examination, I note that the authors’ revision has not substantively addressed these methodological critiques. The response largely reiterates the original methodology without providing new justification or implementing the suggested controls. For instance, while it is true that population-specific FST is sometimes used alongside other methods, its standalone application here is insufficient. Similarly, while window-based approaches like the WZA can be informative, they are typically followed by finer-scale validation to pinpoint causal variants, a step that is absent in the current analysis (refer to Methodological paper of Booker et al. 2021).

Additional Concern: Throughout the manuscript, the authors use the term "adaptive potential" to describe their findings. This terminology is not supported by the presented evidence, which is based solely on genetic association with environmental variables. Without accompanying data on fitness consequences or functional validation, "adaptive potential" is an overstatement. A more precise term, such as "climate-associated genetic variation" or "candidate loci for local adaptation,"

would be more appropriate and scientifically accurate.

Reviewer #4

(Remarks to the Author)
The ms supports the idea that genetically diverse refugial populations, which likely experienced repeated adaptation to novel environments and climates, are more important for the future adaptive capacity of the species than the less diverse populations in the newly colonized areas. This is a very plausible scenario.
The revised ms is now streamlined and the ideas and questions in the intro and discussion are clearly formulated.
General comment:
As far as I understand, ecological modelling was confined to the present distribution range of the barn owl in Europe and North Africa. As a consequence, estimated habitat diversity appears lower for the last glacial maximum (LGM) than today. I think the authors should clarify why they think that a more southern distribution during the ice ages and after the LGM is unlikely.
Specific comments:
In the methods it should always be clearly described whether the measures are calculated window-wise (i.e. for each window separately) or genome-wise (e.g. at l. 588, ...).
l. 1035: "AG" to "AE"
table 1: when there was no resampling, no SD was calculated; replace SD= "0" maybe by "-".
l. 530: shift text in brackets to the right place.
l. 593: replace #SNPs of filtered set by correct number.
l. 557: insert "by".
GO analysis: the analysis could be adjusted for gene length (e.g. as in GOfuncR), because GO terms with predominantly large genes are more likely tagged by genomic selection signals than terms with small genes. Here with no significant results, I don't think this adjustment is important.

Version 2:

Reviewer comments:

Reviewer #2

(Remarks to the Author)
The authors have supplemented the population-specific FST analysis with Sweed analysis and have tested the sensitivity of different WZA window sizes. I have no further comments on the current version

Open Access This Peer Review File is licensed under a Creative Commons Attribution 4.0 International License, which permits use, sharing, adaptation, distribution and reproduction in any medium or format, as long as you give appropriate

credit to the original author(s) and the source, provide a link to the Creative Commons license, and indicate if changes were made.
In cases where reviewers are anonymous, credit should be given to 'Anonymous Referee' and the source.
The images or other third party material in this Peer Review File are included in the article’s Creative Commons license, unless indicated otherwise in a credit line to the material. If material is not included in the article’s Creative Commons license and your intended use is not permitted by statutory regulation or exceeds the permitted use, you will need to obtain permission directly from the copyright holder.
To view a copy of this license, visit https://creativecommons.org/licenses/by/4.0/

Dear Mr Corval,

Your manuscript entitled "Genetically diverse populations hold the keys to climatic adaptation: a lesson from a cosmopolitan raptor" has now been seen by 3 referees, whose comments are appended below. You will see from their comments copied below that while they find your work of considerable interest, they have raised important concerns that must be addressed. In light of these comments, we cannot accept the manuscript for publication but would be interested in considering a revised version that addresses these concerns.

We therefore invite you to revise and resubmit your manuscript, taking into account the points raised. In particular, detail and clarity should be improved throughout the manuscript, but specifically in the Results (gene ontology analysis) and Methods (selection detection and genotype-environment association) sections, as suggested by Reviewers. Please note that we do not impose word limits and all methods should be described in enough detail so that they may be reproduced. Please also carefully consider whether the chosen title accurately reflects the presented results and modify if necessary.

Please highlight all changes in the manuscript text file and provide a point-by-point response to all comments from all reviewers.

We are committed to providing a fair and constructive peer-review process. Do not hesitate to contact us if you wish to discuss the revision in more detail or if there are specific requests from the reviewers that you believe are technically impossible or unlikely to yield a meaningful outcome.

At the same time, we ask that you ensure your manuscript complies with our editorial policies. Specifically:
For all graphs depicting a single point value (e.g., mean) with error bars, you must add individual data points or convert the graph to a boxplot or dot-plot to show data distribution.

It's mandatory to provide access to the numerical source data for graphs and charts either through a repository or by providing the data in a Supplementary Data file (in excel format).

All blots/gels must be accompanied by size markers in every figure panel. Uncropped and unedited blot/gel images must be included as Supplementary Figure(s) in the Supplementary Information pdf.

Please ensure that you have complied with the data deposition policies at the Nature Portfolio, please see, here.

Please ensure that you have complied with our policies on research involving animals and humans, see, here

Please follow the ARRIVE guidelines for reporting animal experiments. Please fully complete an ARRIVE checklist including both the essential and recommended set of items (adding information to the manuscript where needed) and upload this with your revised manuscript.

Please also see our revision checklist for guidance on formatting the manuscript and complying with our policies. A comprehensive guide to our formatting requirements for final submissions is also available for your reference here.

When submitting the revised version of your manuscript, please pay close attention to our Digital Image Integrity Guidelines.

We would like to receive your revision within 3 months but appreciate that every situation is unique. We look forward to receiving your revised manuscript when it is ready and will not enforce a hard deadline on this revision.

Please do not hesitate to contact me if you have any questions or would like to discuss these revisions further. We look forward to seeing the revised manuscript and thank you for the opportunity to review your work.

Best regards,

Michele Repetto, PhD
Associate Editor
Communications Biology
orcid.org/0000-0001-5583-7287

On behalf of

Yi-Jyun Luo, PhD
Editorial Board Member
Communications Biology
orcid.org/0000-0002-3418-3146

Reviewer #1 (Remarks to the Author):
see also attached file for additional comments

This paper provides a valuable contribution on new aspects related to genomic adaptation. I found it good overall and made several suggestions onto the manuscript's PDF. The manuscript's main message is that extant genomic diversity at the core of species' distributions is key to fuel genomic adaptation to climate change, and this is a very relevant point. A very positive aspect of this manuscript is that GEA analyses were performed only in parts of the genome that were found independently to be under selection, a logical approach that sometimes is not practiced in genomic adaptation studies. Overall, the authors do a reasonably good job convincing the reader that their data favors the hypothesis that genomic diversity in refugial areas served as the basis for climate adaptation in newly colonized areas. However, I highlighted across the manuscript's PDF several points that need to be addressed by the authors to improve their work's quality, clarity, and consistency. Finally, I suggest excluding "cosmopolitan raptor" from the paper's title, since the study in focused in the western palearctic, not throughout the world, in fact not taking into account sub-saharian populations of the same species (for more on this refer to my comments onto the PDF).

[R] We thank the reviewer for their thorough reading of our manuscript and for the valuable comments provided. We have now incorporated the suggested changes from the annotated PDF (now listed below) into the revised manuscript. Additionally, we have shortened the title to improve clarity and conciseness.

Line 61 This claim is confusing: sure, they assume that they are adapted to current conditions, but it's not assumed that they cannot evolve. The idea is just to project future offsets based on what's know today. Perhaps a better way to wording this is that these studies fall short from using standing genomic variation to add that as a new layer of information when estimating maladaptation.

[R] We agree with the reviewer’s comment and have revised the text accordingly to improve clarity.

See Line 60: “However, while such predictions provide valuable insights into potential risks, they primarily project future offsets based on current genomic and environmental relationships, without incorporating standing genetic variation as an additional layer of information.”

Line 66 Where? Try to put a geographic context here. Tropical areas, for instance, were affected differently during the LGM.

[R] According to the reviewer’s suggestion, we revised the text to give more geographical context and focus on the Western Palearctic.

See Line 66: “The end of the Last Glacial Maximum in the Western Palearctic created contrasting evolutionary contexts, offering a unique opportunity to study how climate change, along with neutral and selective processes, influenced local adaptation.”

Line 76 These three scenarios are not clear. Are they: migration, local adaptation, and a mix of these two? They are to be explained in a better and more straightforward way

[R] We rephrased and improved the explanation of the three main scenarios following a Last Glacial Maximum recolonisation.

See Line 66: “The end of the Last Glacial Maximum in the Western Palearctic created contrasting evolutionary contexts, offering a unique opportunity to study how climate change, along with neutral and selective processes, influenced local adaptation. In the north, temperatures and melting ice caps opened previously unsuitable regions to (re)-colonisation[1–3]. Populations that tracked their ancestral niche required little adaptation, whereas those entering novel environments likely experienced selection favouring individuals best suited to local conditions, promoting adaptation[4]. Yet, as colonisation advanced through repeated migration events, founder effects may have progressively eroded genetic diversity, constraining adaptive potential along the front of colonisation[5]. At the other end of the range, populations that persisted in the south also faced environmental shift, but their higher genetic diversity may have buffered them against change and enhanced their capacity to adapt[6]. Given these contrasting scenarios, where and how local adaptation happens remains elusive and addressing these questions can deepen our understanding of populations’ adaptive potential.”

Line 140 or 74?

[R] We thank the reviewer for pointing this out, we corrected it.

Line 279 Not in Figure S4! Please, identify it correctly.

[R] We thank the reviewer for pointing this out, we corrected it.

Line 302 And apparently also spread to other populations later on (via gene flow?). I believe something along these lines would have to be mentioned to explain in the PCA plot in Figure 4.

[R] We thank the reviewer for this insightful comment. We agree that the presence of the haploblock in other populations is most likely explained by gene flow following its initial rise in frequency in the two peninsulas. However, as the evolutionary history of this region is not the primary focus of our study, we have only briefly revised the main text to acknowledge this hypothesis. Furthermore, we now consistently use the term “haploblock,” as additional evidence should be provided to classify it as an inversion, as noted by Reviewer 2.

See Line 298: “The history of this haploblock remains also to be clarified. The pattern of selection is shared between the two peninsulas that hosted the species during the Last Glacial Maximum. Considering the isolation of the two populations at the time and the reduced connectivity nowadays (see Figure 4 in Cumer et al., 2021 for details)[7] we suppose that this haploblock predates the Last Glacial Maximum, and that independent and recurrent selection drove the increase of its frequency independently in these two populations. The presence of the haploblock in other populations is most likely due to subsequent gene flow after the Last Glacial Maximum, although additional tests are needed to confirm this scenario.”

Line 340 lays

[R] We thank the reviewer for pointing this out, we corrected it.

Line 347 The species has a much greater distribution than just Europe, also occurring throughout Africa. Please inform why you restricted the modelling to Europe, north Africa, and the middle east, not considering sub-saharian Africa and Madagascar.

[R] We thank the reviewer for this comment. We restricted the modelling to Europe, North Africa, and the Middle East because our subsequent genomic analyses were based exclusively on samples from these regions. Including areas such as sub-Saharan Africa or Madagascar would have introduced predictions for populations for which we had no genetic data, making it impossible to link SDM results with genomic analyses. Our goal was to maintain consistency between the ecological and genomic components of the study and have integrated his rationale in the main text.

See Line 347: “We first conducted species distribution modelling (SDM[8]) to identify suitable areas for the species during the Last Glacial Maximum and nowadays in the Western Palearctic. Although the species also occurs in other regions such as sub-Saharan Africa, we restricted our modelling to the Western Palearctic to focus on processes specific to this area.”

Line 365 The IUCN distribution map does not contain actual occurrence points for the species. As far as I understand, you have to use actual occurrence records from the species to estimate any niche models. Please inform where the distribution points were obtained.

[R] We agree with the reviewer that IUCN maps do not represent actual occurrence records, and further clarification was needed in this section of the manuscript. Rather than using actual occurrence records, we simulated potential presence locations across the known range given by IUCN. We have now explained in greater detail how these occurrence points were sampled in the revised manuscript.

See Line 367: “For each model, we randomly sampled 1,000 points within the species’ geographic range as defined by the IUCN distribution map[9], rather than using actual occurrence records. These points represent potential presence locations within the known range and were generated using the spsample() function implemented in the sp R package[10], after cropping the ICUN shapefile to Europe and North Africa.”

Line 497 There are no coordinates in Table S1!

[R] Table S1 is an independent excel supplementary table, where sample information is provided, including coordinates. We kept its legend in the main Supplementary Material document but changed its layout for better readability.

Reviewer #2 (Remarks to the Author):

Guisan et al. combined species distribution modeling and population genomic approaches to investigate climate adaptation in barn owl. The study documents post-LGM expansion into broader climate niches and identifies putative climate-adaptive variants associated with climate variables. They also detected the climate adaptive variants with a segment of genome inversion. Overall, the study is well designed and sampling size is decent. The species distribution modeling components appear robust, however, several concerns regarding the genomic analyses warrant clarification.

My first concern regards selection detection methodology. The reliance of population-specific FST as the primary selection metric is problematic. FST reflects relative genetic differentiation and is confounded by demographic history, e.g., ancestral polymorphism, population structures and nucleotide diversity. I don't think FST is an appropriate to detect selection. Furthermore, FST is a pairwise comparison. Authors did not adequately justify the computational approach for estimating population-specific FST across multiple populations. This information is critical for evaluating the results. I would recommend to use regular apprTOoaches detecting selective sweep, such as Sweed, number of segregating sites by length (nSL) or the standardized integrated haplotype score (iHS) to detect selective signature.

[R] We appreciate the reviewer's concern regarding the use of F_{ST} to detect selection. While F_{ST} is indeed sensitive to demographic history, differentiation-based genome scans remain a long-standing and widely used class of methods for detecting local adaptation when implemented with appropriate null models and variance control[11–14]. In our study, we specifically applied a population-specific F_{ST} framework[15] to attribute excess divergence to focal populations across a multi-population panel. By computing this differentiation index along the genome, we identify regions of the genome that are more divergent than the rest of the genome. Importantly, we interpret outliers as candidate loci rather than definitive targets of selection.

Numerous empirical studies have validated F_{ST} outliers at known adaptive loci across diverse taxa like humans, sticklebacks, or butterflies[12,16–18]. Comparative evaluations further support the utility of F_{ST}-based scans for detecting geographically structured selection, particularly in cases where selection is polygenic or incomplete[19,20].

Our primary aim was to quantify the extent of genomic regions potentially under selection linked to environmental variation. We believe that combining population-specific F_{ST} with redundancy analysis (RDA) (which is independent of population structure) provides a robust and complementary framework to address this question[21].

Therefore, we have chosen to retain our current approach, while emphasising in the revised manuscript that haplotype-based methods (e.g., iHS, nSL) could be valuable in future work to further characterise the nature and timing of selection in specific regions.

See Line 275: "Nevertheless, haplotype-based methods (e.g., iHS, nSL) could be valuable in future work to further characterize the nature and timing of selection in specific regions."

My second concern regarded genotype-environment association analysis. RDA-based GEA is designed to detect individual SNPs, while the WZA analysis groups linked SNPs. In the submission, 100-kb window size was used for WZA analysis, which appears arbitrary without reference to LD decay patterns (WZA recommendation). This large window size could

artificially inflate lineage signals. Furthermore, the requirement for overlap between GEA-detected variants and FST outliers also lacks the theoretical justification. GEA itself is aimed to detect climate-adaptive genetic variants. FST is to estimate relative genetic differentiation. I can't see the login in this combination.

[R] We appreciate the reviewer's concern regarding the genotype–environment association (GEA) analysis and the use of window-based statistics. While RDA-based GEA is designed to detect associations at the SNP level, combining it with window-based approaches such as WZA allows to reduce noise and identify genomic regions rather than isolated markers[22,23]. The 100-kb window size was chosen based on prior empirical work in this species[7], where it effectively captured linkage blocks without excessive smoothing (see figure below). We now clarify this rationale in the revised manuscript.

See Line 558: "We computed the weighted-Z statistics on the same windows as population-specific FST (size of 100 kbp, step of 20 kbp). This consistency enables direct comparison between the two approaches and aligns with previous empirical studies in this species[7]. While alternative window sizes were considered, we chose the 100 kbp window as a compromise to reduce stochastic noise while retaining the resolution necessary to detect meaningful signals."

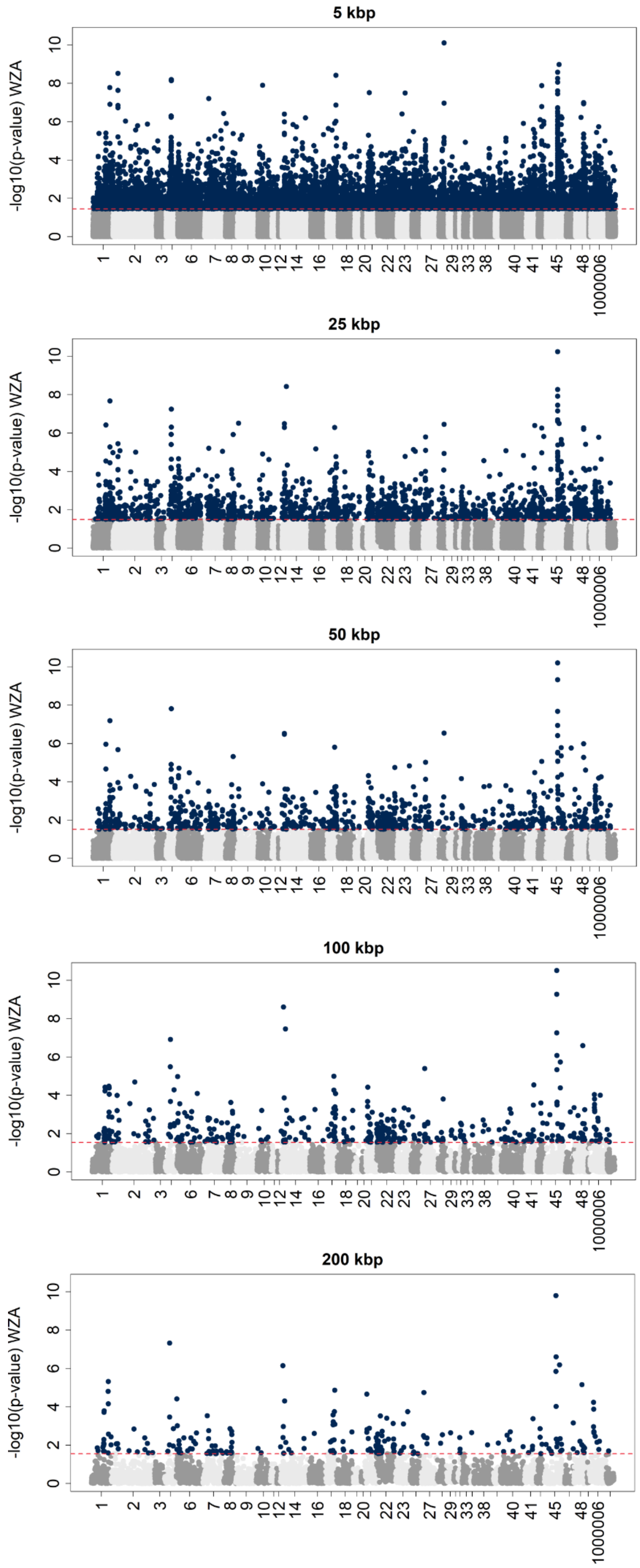

Figure R1. Genome-wide distribution of WZA scores for different window sizes. Dark blue dots represent windows with z-scores more than two standard deviations above the mean. Note that the windows shown here are non-overlapping, whereas overlapping windows are used in the main text.

Regarding the combination of GEA and F_{ST} outliers, we respectfully disagree with the notion that this lacks theoretical justification. GEA identifies loci associated with environmental gradients, and F_{ST} highlights loci with elevated differentiation; loci identified by these 2 unrelated approaches are thus sound candidates for positions under the influence of local adaptation, as acknowledge by the two other reviewers. Such combination helps filter out false positives from either method alone and has been widely applied in landscape genomics[21,24]. Once again, our goal was not to detect selective sweeps per se, but to identify genomic regions potentially shaped by environmental selection. The combination of RDA and population-specific F_{ST} provides complementary perspectives, one based on environmental gradients, the other on spatial genetic structure. We believe this integrative framework is appropriate for our study system and question. This is now better emphasised in the main text.

See Line 273: "Our approach, which integrates differentiation-based and environment-association methods, is recommended to detect signals of adaptation in natural populations and provides complementary evidence for selection[21]."

And Line 570: "Because our study aimed to detect genomic regions potentially shaped local climatic conditions, we defined outlier windows as those overlapping between the genome scans (see Detection of traces of selection in each population section) and the Genotype-Environment Association analysis (see Weighted-Z Analysis section). Such integrative approach that combines GEA with differentiation-based metrics such as FST strengthen inference by reducing false positives and identifying loci under spatially varying selection[21]."

My third concern referred inversion characterization. The claim of inversion requires strong evidence rather than local PCA alone. Supplemental analyses should include recombination rate suppression, LD pattern across the putative inversion.

[R] We agree with the reviewer that claiming the presence of an inversion requires more than local PCA evidence. We have gathered additional support for our hypothesis, including both indirect and direct lines of evidence. Indirectly, the region shows elevated linkage disequilibrium (LD), a local PCA with three distinct clusters along the first axis, and increased heterozygosity in individuals from the central cluster, patterns consistent with a segregating inversion. Directly, we have also examined long-read sequencing data, which further supports the presence of structural variation in this region.
However, the full characterization of the inversion (including its breakpoints, recombination suppression, and fitness effects) requires dedicated analyses beyond the scope of this study. These will be addressed in a future publication.

To avoid overstatement and align with the reviewer's recommendation, we have revised the manuscript to refer to this region as a "haploblock" rather than an inversion. This terminology reflects the observed genomic structure while remaining agnostic about the underlying mechanism.

My last concern regarded overstatements in Discussion, in particular regarding interpretations about gene ontology, and climate adaptation of inversion. For example,
Discussion:
"Line 255-272. I think this discussion is too speculative regarding that authors only based a few gene ontology terms. These terms are quite broad and basic functions. For example, in

L261 “This finding aligns with previous research showing that pathogens and infectious diseases exert strong selective pressures on both humans and birds40,41”
I don’t think you can infer this from your results, with only based a few identified immune related genes. Immune can response many ecological factors, not only pathogens and infectious disease
“Line 263 temperature and rainfall significantly influence pathogen community composition42,4”
Again, temperature and rainfall not only influences pathogen community
“Line 268. This result was consistent with Bergmann’s 269 rule which predicts that body sizes of warm-blooded vertebrates negatively correlate with temperature, leading to smaller body sizes in warmer climates45,46 270.”
You can’t infer a Bergmanns’ evidence only based on the gene ontology enriched by genes related developmental process and morphogenesis.

[R] We agree with the reviewer’s comment and made major changes in the section about the genes functions to avoid any overstatements.

See Line 268: “Across the species’ range, we detected 550 genes located within regions significantly associated with environmental conditions and under selection. Despite no significant enrichment for specific pathways, the extent of the genome impacted by the climate reinforces the view that local adaptation is a complex, polygenic process involving numerous loci distributed across the genome.”

“Line 291-293 Briefly, considering the genomic disruption that breakpoints can cause, as well as the fact that nearly suppressing recombination will prevent purging of deleterious alleles in the inverted haplotype, it is unlikely for a newly appeared inversion to persist and spread in a population51”

I think inferring inversion only by local PCA is not enough. A start-of-art method is to check recombination rate. I did not find recombination estimation in Results, but in Discussion authors referred suppression recombination. Did I miss this in Results? Besides, even the inversion occurred, how it related to climate adaptation?

[R] We thank the reviewer for pointing out this inconsistency. As previously mentioned, we have gathered additional evidence beyond the local PCA indicating that this region is non-recombining and likely represents a region with disturbed recombination. To support this, we have included in the supplementary material (Supplementary Figure 13) a map of pairwise R^2 values between SNPs in this region, calculated for all individuals and separately for individuals heterozygous for this haplotype only. This figure show that, focusing on heterozygous individuals, SNPs are in strong linkage (high R^2) and do not recombine. In contrast, when including homozygous individuals only, recombination appears possible, as reflected by the lower R^2 values. As noted above, we now refer to this region as a haploblock.

We have now included details about the R^2 computation and its interpretation in the material and method section.

See Line 512: “Additionally, we randomly sampled 50,000 SNPs among this region and computed R^2 among heterozygous individuals for this haploblock, using the SNPRelate R package[25].”

And in the result section.

See Line 230: “Consistent with this clustering, linkage disequilibrium (R^2) among variants in this region was particularly high in heterozygous individuals (i.e., middle cluster), while it was comparatively lower when computed across all individuals (Supplementary Figure 13), reinforcing the interpretation of reduced recombination within these haploblocks.”

The direct link between this haploblock (or inversion) and climate remains unresolved. However, numerous studies have demonstrated associations between chromosomal inversions and local adaptation. For example, inversions have been implicated in maintaining ecotypic differentiation in deer mice, where a large inversion underlies variation in tail length and coat colour between forest and prairie ecotypes (Hager et al., 2022). In Atlantic cod, several megabase-scale inversions contribute to divergence in life-history traits and migratory behaviour between ecotypes across the North Atlantic (Berg et al., 2017). Inversions have also been associated with climatic clines without direct evidence of the underlying traits, such as in the seaweed fly Coelopa frigida, where inversion frequencies form parallel latitudinal clines across continents, suggesting adaptation to thermal gradients (Nicolas et al., 2025; Mérot et al., 2021). Similarly, in marine snails (Littorina spp.), some inversion clines correlate with environmental gradients (Le Moan et al., 2024; Faria et al., 2024)

“Line 303-309 A key question is the origin of adaptive alleles and given the pattern of genomic traces of local adaptation we observed, we propose that standing variation played a crucial role in this process54,55. One possibility for local adaptation to occur is de novo mutations bringing new favourable alleles in the different populations54,56. Considering the low mutation rate57,58 and the even lower probability of a mutation being both advantageous and occurring in the right environmental context59, this mechanism is unlikely to fully explain the extent of genomic regions involved in local adaptation (Figure 2)”.

I don’t think this is particular true. Regarding the method you used to infer climate adaptation, FST and genotype-environment association, you can only identify standing variation. This sentence seems not necessary as your experimental design was not able to identify de novo mutation.

[R] We appreciate the reviewer’s comment and agree that our methods (population-specific F_{ST} and GEA) are not designed to directly identify *de novo* mutations, and de facto do not consider mutations at low frequency. However, we note that adaptive *de novo* mutations that have risen to moderate or high frequency due to selection can, in principle, be detected by these methods.
While we cannot determine the origin of individual alleles, the widespread and structured nature of the signals we observe (across multiple populations and environmental gradients) makes standing variation a more plausible explanation than repeated, independent de novo mutations.

This reasoning is supported by theoretical and empirical studies (e.g., Barrett & Schluter 2008), which show that standing variation often plays a key role in local adaptation, especially when mutation rates are low and selection acts on pre-existing alleles.

We have revised the manuscript to clarify that our inference about standing variation is based on biological plausibility and the observed patterns, rather than direct evidence of allele origin.

See Line 306: "A key question when studying local adaptation is the origin of adaptive alleles, either arising *de novo* or from standing genetic variation. Given the pattern of genomic signals we observed, standing variation is likely to have played a central role in this process[26,27]. While *de novo* mutations can also contribute to adaptation by introducing new beneficial alleles[26,28], their impact is limited by low mutation rate[29,30] and the even lower probability of such mutations being both advantageous and occurring in the right environmental context[31]. The reasoning is consistent with theoretical and empirical studies[21,27], which emphasise that standing genetic variation is often the primary substrate for local adaptation, especially under spatially heterogeneous selection."

Methods
Line 419-424. Please provide specific information about your vcf calling and filtering. What is hard-filtering approach? Not every author would go to check Cumer et al. 2022.

[R] We thank the reviewer for pointing this out. We agree that relying solely on a previous publication[3] may not provide sufficient clarity for all readers. In response, we have now included a standalone description of our VCF calling and filtering pipeline in the revised manuscript. Specifically, we clarify the hard-filtering approach used, including the thresholds applied for variant quality, depth, missingness, and minor allele frequency. This ensures transparency and reproducibility, and we hope it adequately addresses the reviewer's concern.

For the update version, see Line 425.

Reviewer #3 (Remarks to the Author):

Review of the Article:
Genetically diverse populations hold the keys to climatic adaptation: a lesson from a cosmopolitan raptor

The authors studied local adaptation using the European barn owl, applying Species Distribution Modelling and characterizing climatic conditions in relation to the Last Glacial Maximum. The authors combined a genome scan for selection and a Genotype-Environment Association (GEA) analysis to identify genomic regions and genes potentially involved in the local adaptation of the different populations. I found the methodology particularly well developed and applied for the research question, and I consider that the study would be of great interest to Communications Biology's readers.

I only have 2 main concerns. One is a (1) suggestion to improve the clarity of the results, and the other is a (2) comment in relation to the Gene Ontology Enrichment (GOE) analysis. Some minor comments can be found below, ordered by sections, and specific lines are signalled with L.(#line).

Major comments:
(1) It is somewhat difficult to identify the main message while reading the Results section. The individual parts appear somewhat scattered and disconnected at first glance, and it requires additional effort from the reader to grasp the overarching link. I suggest restructuring the Results and Discussion section with a clearer hierarchical organization, moving from the general (highlighting the main findings) to the specific details. From my perspective, the main point is the genomic signal of selection that the authors found linked to climate, but I see the whole description of the climate as an accessory part to get there.

[R] We thank the reviewer for their thoughtful comment regarding the structure of the Results section. We understand the concern about the flow of ideas and agree that clearer transitions could help readers follow the logic more easily. However, we chose to present the climate reconstruction first, followed by genomic analyses, because our central hypothesis is that climate change preceded and potentially drove local adaptation. If climatic conditions had remained stable since the Last Glacial Maximum, there would be little rationale for expecting genomic signatures of adaptation driven by climate change.
To improve clarity and coherence, we have revised the Results section to better link the climatic context with the subsequent genomic findings. Specifically, we added transitional sentences that explicitly connect the observed climatic shifts to the rationale for investigating genomic adaptation, thereby reinforcing the logical progression of our argument.

For sentences smoothening the results section and linking the different sections, see Lines 110, 148, 195 and 214.

(2) The Gene Ontology Enrichment analysis appears to use a very broad approach, which results in outputs that are too general and difficult to interpret. I recommend refining the analysis with a more specialized avian annotation. For example, in ShinyGo v0.8106 the Gallus gallus genome could be selected as a reference, or alternatively, the authors could build a

customized annotation tailored to their dataset. It is also not clear if the discussion about the found GO terms in the main text is based on statistically significant results or not, or if they were controlled by multiple testing or not? Finally, I suggest improving the readability of the supplementary material to ensure transparency and reproducibility. For example, the names and headers of the tables can be clearer, the headers of the columns should be meaningful and self-explanatory.

[R] We appreciate the reviewer's insightful comments regarding the Gene Ontology enrichment analysis and agree that the previous version was challenging to interpret.

In response, we have implemented the following changes:

- Refined the GO analysis by focusing on the overall list of candidate genes rather than population-specific subsets, which previously added complexity without improving interpretability.
- Incorporated *Gallus gallus* as the reference genome in ShinyGO, as suggested. This change did not alter the results.
- Improved the supplementary material for clarity and reproducibility. Table names and column headers have been revised to be more descriptive and self-explanatory.

These changes were incorporated in the material and methods section.

See Line 583: “We performed one GOE using all genes detected in at least one population. As a baseline set of genes against which we compared the observed enrichment signature, also called the background list, we used all the genes annotated in the barn owl genome that could have been detected with our 52’429 non-overlapping windows from the genome scans or WZA. For each analysis, we used the default pathway databases, namely KEGG (Kyoto Encyclopedia of Genes and Genomes), as well as the GO Biological Process database. GOEs were ran using both the human (*Homo sapiens*) and the chicken (*Gallus gallus*) as reference species.”

And in the result section.

See Line 189: “Within the 550 extracted genes, Gene Ontology Enrichment (GOE) analysis produced no significant terms with the chicken reference (Supplementary Table 4). Using the human reference yielded a single significant term, namely pancreatic secretion (Supplementary Table 5).”

Additionally, we have substantially revised the discussion of gene functions to avoid overinterpretation.

See Line 267: “[…] we found genomic evidence of local adaptation to climatic variables (humidity and temperature) in all populations. Across the species’ range, we detected 550 genes located within regions significantly associated with environmental conditions and under selection. Despite no significant enrichment for specific pathways, the extent of the genome impacted by the climate reinforces the view that local adaptation is a complex, polygenic process involving numerous loci distributed across the genome.”

Minor comments:
Title
1. Consider making the title more direct. For example, replacing “Genetically diverse populations hold the keys to” with “Genetic diversity is the key for”. Other options: “Genetic diversity underpins past and future climate adaptation in the barn owl”, or “Genetic diversity as the foundation of barn owl resilience to climate change”, etc.

[R] We thank the reviewer for this suggestion. We changed the title by removing the second part, making it more direct and more general.

Abstract and Introduction
2. Please avoid the unnecessary use of the word “demonstrate”, instead, use a softer one like “show” unless there is really strong evidence for a statement.

[R] We thank the reviewer for this suggestion. We switched to more moderate wording throughout the manuscript.

3. L 66 I suggest choosing one consistent style to mention the Last Glacial Maximum (LGM) across the text from this line. I think keeping the whole words would be preferred, considering the broader audience of this journal.

[R] We thank the reviewer for this suggestion. We changed LGM into Last Glacial Maximum to be consistent throughout the manuscript.

Results
4. See major comments

[R] See our answers to major comments.

Discussion
5. Same as in the Introduction, please replace the word “demonstrated” with a more moderate one.

[R] We thank the reviewer for this suggestion. See the answer above.

6. L. 338 I think the article would benefit here from a bit of highlighting the importance of the Barn owl for the European ecosystems. This is something that could also be included in the introduction as part of the importance of the study. I mean, what would be the risk if the Barn owl didn’t have such adaptive capacity?

[R] We thank the reviewer for this comment. We appreciate your suggestion to highlight the ecological importance of the European barn owl. However, our aim with this study was not to focus on species-specific conservation aspects, but rather to use the barn owl as a model system to explore broader ecological and evolutionary questions. Emphasizing its role in European ecosystems might narrow the scope and shift the focus toward species-specific implications, which is not the primary objective of our work.

To clarify this, we have revised the introduction to better reflect the rationale behind choosing the barn owl for its suitability as a study system to address general questions about adaptive capacity and resilience across diverse taxa. We believe this framing helps maintain the broader relevance of our findings.

See Line 79: “The barn owl (Tyto alba), a nonmigratory raptor distributed across the Western Palearctic, offers a powerful system to investigate the patterns and processes of local adaptation, a theme central to many species facing environmental heterogeneity.”

And Line 92: “[…] make this species an attractive model to test and quantify the extent and location of local adaptation in face of climatic variation, a question of relevance across many taxa.”

In line with the reviewer’s comment, we also modified the last paragraph of the discussion to emphasize even more the broader implications of our findings about the adaptive capacity and resilience across multiple taxa and not only the barn owl.

See Line 329: “This suggests that adaptation in refugial populations is not static but a recurrent and dynamic process, a pattern likely shared across many taxa that persisted in historical refugia.”

And Line 339: “[…] our study lays the groundwork for a deeper understanding of the genomic foundations of adaptative potential, a key component of resilience across taxa. Future research will be essential to uncover the mechanisms that enable diverse species to persist, adapt, and thrive amid accelerating environmental change.”

Materials and Methods
7. Ok, only see my comment about the Gene Ontology Enrichment (GOE) analysis

[R] See our answers to major comments.

1. Hewitt, G. The genetic legacy of the Quaternary ice ages. *Nature* **405**, 907–913 (2000).
2. Hortal, J. *et al.* Ice age climate, evolutionary constraints and diversity patterns of European dung beetles: Ice age determines European scarab diversity. *Ecol. Lett.* **14**, 741–748 (2011).
3. Cumer, T. *et al.* Landscape and Climatic Variations Shaped Secondary Contacts amid Barn Owls of the Western Palearctic. *Mol. Biol. Evol.* **39**, msab343 (2022).
4. Kawecki, T. J. & Ebert, D. Conceptual issues in local adaptation. *Ecol. Lett.* **7**, 1225–1241 (2004).
5. Slatkin, M. & Excoffier, L. Serial Founder Effects During Range Expansion: A Spatial Analog of Genetic Drift. *Genetics* **191**, 171–181 (2012).
6. Svenning, J.-C., Eiserhardt, W. L., Normand, S., Ordonez, A. & Sandel, B. The Influence of Paleoclimate on Present-Day Patterns in Biodiversity and Ecosystems. *Annu. Rev. Ecol. Evol. Syst.* **46**, 551–572 (2015).
7. Cumer, T. *et al.* Genomic basis of insularity and ecological divergence in barn owls (Tyto alba) of the Canary Islands. *Heredity* **129**, 281–294 (2022).
8. Guisan, A., Thuiller, W. & Zimmermann, N. E. *Habitat Suitability and Distribution Models: With Applications in R*. (Cambridge university press, Cambridge, United Kingdom, 2017).
9. BirdLife International. IUCN Red List of Threatened Species: Tyto alba. *IUCN Red List Threat. Species* https://www.iucnredlist.org/en (2016).
10. Pebesma, E. *et al.* sp: Classes and Methods for Spatial Data. (2025).
11. Beaumont, M. A. & Nichols, R. A. Evaluating Loci for Use in the Genetic Analysis of Population Structure. *Proc. Biol. Sci.* **263**, 1619–1626 (1996).
12. Weir, B. S., Cardon, L. R., Anderson, A. D., Nielsen, D. M. & Hill, W. G. Measures of human population structure show heterogeneity among genomic regions. *Genome Res.* **15**, 1468–1476 (2005).
13. Holsinger, K. E. & Weir, B. S. Genetics in geographically structured populations: defining, estimating and interpreting FST. *Nat. Rev. Genet.* **10**, 639–650 (2009).

14. Foll, M. & Gaggiotti, O. A Genome-Scan Method to Identify Selected Loci Appropriate for Both Dominant and Codominant Markers: A Bayesian Perspective. *Genetics* **180**, 977–993 (2008).

15. Weir, B. S. & Goudet, J. A Unified Characterization of Population Structure and Relatedness. *Genetics* **206**, 2085–2103 (2017).

16. Akey, J. M., Zhang, G., Zhang, K., Jin, L. & Shriver, M. D. Interrogating a High-Density SNP Map for Signatures of Natural Selection. *Genome Res.* **12**, 1805–1814 (2002).

17. Hohenlohe, P. A. *et al.* Population Genomics of Parallel Adaptation in Threespine Stickleback using Sequenced RAD Tags. *PLoS Genet.* **6**, e1000862 (2010).

18. Nadeau, N. J. *et al.* Genomic islands of divergence in hybridizing *Heliconius* butterflies identified by large-scale targeted sequencing. *Philos. Trans. R. Soc. B Biol. Sci.* **367**, 343–353 (2012).

19. Lotterhos, K. E. & Whitlock, M. C. The relative power of genome scans to detect local adaptation depends on sampling design and statistical method. *Mol. Ecol.* **24**, 1031–1046 (2015).

20. Lotterhos, K. E. & Whitlock, M. C. Evaluation of demographic history and neutral parameterization on the performance of F_{ST} outlier tests. *Mol. Ecol.* **23**, 2178–2192 (2014).

21. Rellstab, C., Gugerli, F., Eckert, A. J., Hancock, A. M. & Holderegger, R. A practical guide to environmental association analysis in landscape genomics. *Mol. Ecol.* **24**, 4348–4370 (2015).

22. Lotterhos, K. E. *et al.* Composite measures of selection can improve the signal-to-noise ratio in genome scans. *Methods Ecol. Evol.* **8**, 717–727 (2017).

23. Forester, B. R., Lasky, J. R., Wagner, H. H. & Urban, D. L. Comparing methods for detecting multilocus adaptation with multivariate genotype–environment associations. *Mol. Ecol.* **27**, 2215–2233 (2018).

24. Hoban, S. *et al.* Finding the Genomic Basis of Local Adaptation: Pitfalls, Practical Solutions, and Future Directions. *Am. Nat.* **188**, 379–397 (2016).

25. Xiuwen Zheng [Aut, C. SNPRelate. Bioconductor https://doi.org/10.18129/B9.BIOC.SNPRELATE (2017).
26. Bomblies, K. & Peichel, C. L. Genetics of adaptation. *Proc. Natl. Acad. Sci.* **119**, e2122152119 (2022).
27. Barrett, R. & Schluter, D. Adaptation from standing genetic variation. *Trends Ecol. Evol.* **23**, 38–44 (2008).
28. Chan, Y. F. *et al.* Adaptive Evolution of Pelvic Reduction in Sticklebacks by Recurrent Deletion of a *Pitx1* Enhancer. *Science* **327**, 302–305 (2010).
29. Smeds, L., Qvarnström, A. & Ellegren, H. Direct estimate of the rate of germline mutation in a bird. *Genome Res.* **26**, 1211–1218 (2016).
30. Bergeron, L. A. *et al.* Evolution of the germline mutation rate across vertebrates. *Nature* **615**, 285–291 (2023).
31. Rees, J. S., Castellano, S. & Andrés, A. M. The Genomics of Human Local Adaptation. *Trends Genet.* **36**, 415–428 (2020).

Reviewer #2:

This is my second review of this submission. In my initial review, I raised two primary methodological concerns that are central to the validity of the authors' conclusions.

First, the continued reliance on population-specific FST as the primary metric for detecting selection remains problematic. While FST is a useful measure of genetic differentiation, it is strongly confounded by demographic history—including ancestral polymorphism, population structure, and local variation in nucleotide diversity. Consequently, high FST values alone are not robust signatures of selective sweeps. I had recommended supplementing or replacing this approach with established selection detection methods—such as SweeD, nSL, or iHS—which are designed to distinguish selective signals from demographic noise.

[R] In our previous revision, we acknowledged that FST may be confounded by other factors but we think, in line with the opinion from other reviewers that the combination the GEA is a valid approach to identify regions linked with the climate. Moreover, multiple studies now shows that methods directly designed to detect sweeps are also affected by migration, demographic history, sweep type (hard vs soft), and sweep stage and sample size (Koropoulis et al., 2020; Vatsiou et al., 2016). Nonetheless, we have now included a SweeD analysis in the manuscript. This approach allowed to confirm that 159 of the 270 regions identified in our previous scan harbour signal of selective sweeps.
See our revisions Line 541:
"To further confirm the signal identified by the population-specific FST approach, we used the Likelihood-Based Detection of Selective Sweeps implemented in SweeD[99], on a window-basis. Sweed was run for each population independently as well as across all populations. SweeD grid size parameter (-grid) was adjusted based on chromosome length to obtain a ~10kbp resolution along the genome and using folded mode (-folded) since variants were not polarised. We considered a region as an outlier in each population and across populations if its likelihood was equal to or higher than 2 standard deviations from the mean."

And line 189:
"Among these 270 regions, 159 presented signs of ongoing selective sweep (Supplementary Figure 8 and 9)."

Second, the window-based climate association analysis (using 100 kb windows) is susceptible to identifying large false-positive associations, as large windows inevitably contain many SNPs unlinked to the adaptive trait. I previously suggested a more targeted follow-up analysis, such as testing individual climate-associated SNPs identified within these candidate windows, to improve specificity.

[R] We thank the reviewer for this comment. While reading the first review, we acknowledge that the 100 kb window size was mentioned, and we have now included the results of our analysis of the consistency of the results depending on window size in the supplementary figures of the paper (see supplementary figure S19).
Regarding the SNP-based analysis. By definition, our detection of climate-associated regions was based on SNP-level analyses using an RDA approach. These SNP-specific signals were then aggregated using

the WZA method since it "outperformed single-SNP-based approaches [...] particularly when a small number of individuals or demes were sampled" (Booker et al., 2024). Repeating another SNP-based analysis therefore seems redundant, but we now provide a table including significant SNP from the RDA and associated p-values within the candidate regions as a supplementary table S3.
We now point to this table line 601: “we chose the 100 kbp window as a compromise to reduce stochastic noise while retaining the resolution necessary to detect meaningful signals (Supplementary Figure 19).”

Upon re-examination, I note that the authors’ revision has not substantively addressed these methodological critiques. The response largely reiterates the original methodology without providing new justification or implementing the suggested controls. For instance, while it is true that population-specific FST is sometimes used alongside other methods, its standalone application here is insufficient. Similarly, while window-based approaches like the WZA can be informative, they are typically followed by finer-scale validation to pinpoint causal variants, a step that is absent in the current analysis (refer to Methodological paper of Booker et al. 2021).

[R] We agree with the reviewer that our previous revision didn’t integrate new analyses, however we did we provided details about our reasoning on the combination of population specific FST and RDA to achieve the aims of the paper.

We want to stress here that the aim of this paper was not to identify causal SNPs responsible to the adaptation in a given population to a given environmental variable, but quantify the extend of local adaptation driven by standing variant, as stated line 67 of the manuscript: “[...] a key challenge remains understanding whether the existing genetic diversity can fuel adaptation to new climate, a question our study seeks to answer”.
Thus using finer scale analyses to detect causal variants is beyond the scope of this study. That being said, and in line with our answer to the previous comment, we now provide the RDA q-value for all the SNPs in the regions we identified.

Additional Concern: Throughout the manuscript, the authors use the term "adaptive potential" to describe their findings. This terminology is not supported by the presented evidence, which is based solely on genetic association with environmental variables. Without accompanying data on fitness consequences or functional validation, "adaptive potential" is an overstatement. A more precise term, such as "climate-associated genetic variation" or "candidate loci for local adaptation," would be more appropriate and scientifically accurate.

[R] We thank the reviewer for this comment. We have now changed the instances of “adaptive potential” that were related to our finding's with “candidate loci for local adaptation”.
See line 33 and 111.

Reviewer #4:

The ms supports the idea that genetically diverse refugial populations, which likely experienced repeated adaptation to novel environments and climates, are more important for the future adaptive capacity of the species than the less diverse populations in the newly colonized areas. This is a very plausible scenario.
The revised ms is now streamlined and the ideas and questions in the intro and discussion are clearly formulated.

General comment:

As far as I understand, ecological modelling was confined to the present distribution range of the barn owl in Europe and North Africa. As a consequence, estimated habitat diversity appears lower for the last glacial maximum (LGM) than today. I think the authors should clarify why they think that a more southern distribution during the ice ages and after the LGM is unlikely.

[R] We thank the reviewer for this valuable comment. We indeed restricted the ecological modelling to a region encompassing continental Europe and the Mediterranean basin. Importantly, this area includes both currently suitable and unsuitable regions for the barn owl. Our choice was driven by previous population genetic and phylogeographic studies showing that contemporary European barn owls predominantly recolonized Europe from two main glacial refugia located in the Iberian and Italian peninsulas ((Antoniazza et al., 2014; Cumer et al., 2022)).
Because these two southern peninsulas have been identified as the primary sources of postglacial recolonisation, extending the climatic modelling to substantially more southern regions (e.g., deep North Africa or Asia) could bias the inference. Such areas may have been climatically suitable during the LGM but are unlikely to have contributed to the genetic pool of present-day European barn owls, based on current evidence. Including them would therefore introduce habitat suitability signals from populations that do not correspond to the ancestry of the populations under study.
In line with this reasoning, and as shown in Fig. S1, the climatically suitable area for barn owls during the LGM does not extend beyond the boundaries of the region we selected. Thus, the spatial restriction we applied corresponds well to the known biogeographic history of the species and provides the appropriate scale for our ecological modelling.

We have now integrated this rational in the main text, see line 373:
"Although the species also occurs in other regions such as sub-Saharan Africa, we restricted our modelling to the Western Palearctic because previous phylogeographic studies indicate that present‑day European populations mainly recolonised the continent from Iberian and Italian refugia [15]. Extending the modelling further south would therefore introduce regions that were unlikely to contribute to the genetic ancestry of the populations studied."

Specific comments:

In the methods it should always be clearly described whether the measures are calculated window-wise (i.e. for each window separately) or genome-wise (e.g. at l. 588, ...).

[R] We thank the reviewer for this comment. We have now modified the Material and method to clearly state when analyses where SNP-based or Window-based.

l. 1035: "AG" to "AE"

[R] Changed

table 1: when there was no resampling, no SD was calculated; replace SD= "0" maybe by "-

[R] Changed

l. 530: shift text in brackets to the right place.

[R] Done

l. 593: replace #SNPs of filtered set by correct number.

[R] This number is correct. RDA analysis doesn't support missing data, we thus used a restricted set of SNPs, MAF filtered and imputed, see section *Phasing process and evaluation* for details about this procedure.
We have now clarified this rationale in the main text, see line 553:
"Since RDA doesn't support missing data, we used the imputed genotype matrix (phased set of 4'689'284 SNPs, see Phasing process and evaluation section for details) as the response matrix and the bioclimatic variables extracted at each sampling locality for the multiple linear regression."

l. 557: insert "by".

[R] Done

GO analysis: the analysis could be adjusted for gene length (e.g. as in GOfuncR), because GO terms with predominantly large genes are more likely tagged by genomic selection signals than terms with small genes. Here with no significant results, I don't think this adjustment is important.

[R] We thanks the reviewer for pointing out this potential bias. We agree that in our case with no significant results, re-running the analysis woudn't change the message of the paper, however, we now point toward potential biais in our approach in the discussion of the paper, see line 290:

"Among the 550 genes identified in these regions, no significant enrichment for specific pathways was found. We note, however, that GO analyses may be suboptimal, as GO terms composed predominantly of large genes are more likely to be tagged by genomic selection signals than terms containing smaller genes [41], and further work will be needed to identify the specific pathways and genes involved in the local adaptation of populations. Thus, despite the lack of significant pathway enrichment, the extent of the genome impacted by the climate reinforces the view that local adaptation is a complex, [...]"

References

Antoniazza, S., Kanitz, R., Neuenschwander, S., Burri, R., Gaigher, A., Roulin, A., & Goudet, J. (2014). Natural selection in a postglacial range expansion: The case of the colour cline in the European barn owl. *Molecular Ecology*, *23*(22), 5508–5523. https://doi.org/10.1111/mec.12957

Cumer, T., Machado, A. P., Dumont, G., Bontzorlos, V., Ceccherelli, R., Charter, M., Dichmann, K., Kassinis, N., Lourenço, R., Manzia, F., Martens, H.-D., Prévost, L., Rakovic, M., Roque, I., Siverio, F., Roulin, A., & Goudet, J. (2022). Landscape and Climatic Variations Shaped Secondary Contacts amid Barn Owls of the Western Palearctic. *Molecular Biology and Evolution*, *39*(1), msab343. https://doi.org/10.1093/molbev/msab343

Koropoulis, A., Alachiotis, N., & Pavlidis, P. (2020). Detecting Positive Selection in Populations Using Genetic Data. In J. Y. Dutheil (Ed.), *Statistical Population Genomics* (Vol. 2090, pp. 87–123). Springer US. https://doi.org/10.1007/978-1-0716-0199-0_5

Vatsiou, A. I., Bazin, E., & Gaggiotti, O. E. (2016). Detection of selective sweeps in structured populations: A comparison of recent methods. *Molecular Ecology*, *25*(1), 89–103. https://doi.org/10.1111/mec.13360
