# Supplementary material for: Genetically diverse populations hold the keys to climatic adaptation in the Western barn owl (Tyto alba)
Source: Commun Biol. 2026 Jun 3;9:1172. doi: 10.1038/s42003-026-10385-8 (PMC13542081; doi:10.1038/s42003-026-10385-8)
Supplement: Supplementary file 3 [file 42003_2026_10385_MOESM3_ESM.pdf]

Description of Additional Supplementary Files

File name: Supplementary Data 1
Description: Description of the samples used in this study. See additional file.

File name: Supplementary Data 2
Description: SNPs identified by redundancy analysis (RDA) in windows shared between selection scans and the weighted Z-analysis (WZA).

File name: Supplementary Data 3
Description: Gene ontology results with chicken as a reference.

File name: Supplementary Data 4
Description: Gene ontology results with human as a reference.
