# Supplementary material for: Genetically diverse populations hold the keys to climatic adaptation in the Western barn owl (Tyto alba)
Source: Commun Biol. 2026 Jun 3;9:1172. doi: 10.1038/s42003-026-10385-8 (PMC13542081; doi:10.1038/s42003-026-10385-8)
Supplement: Supplementary file 4 [file 42003_2026_10385_MOESM8_ESM.pdf]

nature portfolio

Corresponding author(s): Hugo Corval
Tristan Cumer
Jérôme Goudet

Last updated by author(s): May 11, 2026

Reporting Summary

Nature Portfolio wishes to improve the reproducibility of the work that we publish. This form provides structure for consistency and transparency in reporting. For further information on Nature Portfolio policies, see our Editorial Policies and the Editorial Policy Checklist.

Statistics

For all statistical analyses, confirm that the following items are present in the figure legend, table legend, main text, or Methods section.

n/a	Confirmed	
☐	☒	The exact sample size (*n*) for each experimental group/condition, given as a discrete number and unit of measurement
☐	☒	A statement on whether measurements were taken from distinct samples or whether the same sample was measured repeatedly
☐	☒	The statistical test(s) used AND whether they are one- or two-sided<br>*Only common tests should be described solely by name; describe more complex techniques in the Methods section.*
☐	☒	A description of all covariates tested
☐	☒	A description of any assumptions or corrections, such as tests of normality and adjustment for multiple comparisons
☐	☒	A full description of the statistical parameters including central tendency (e.g. means) or other basic estimates (e.g. regression coefficient) AND variation (e.g. standard deviation) or associated estimates of uncertainty (e.g. confidence intervals)
☐	☒	For null hypothesis testing, the test statistic (e.g. *F*, *t*, *r*) with confidence intervals, effect sizes, degrees of freedom and *P* value noted<br>*Give P values as exact values whenever suitable.*
☒	☐	For Bayesian analysis, information on the choice of priors and Markov chain Monte Carlo settings
☒	☐	For hierarchical and complex designs, identification of the appropriate level for tests and full reporting of outcomes
☐	☒	Estimates of effect sizes (e.g. Cohen's *d*, Pearson's *r*), indicating how they were calculated

Our web collection on statistics for biologists contains articles on many of the points above.

Software and code

Policy information about availability of computer code

Data collection	All data were retrieved from the GenBank Sequence Read Archive Databse, see "Data" section.
Data analysis	MaxEnt v.3.4.3, WorldClim 1.4 database, R v.4.3.2, BWA-MEM v.0.7.15, GATK v.4.1.3, VCFTools v.0.1.14, WhatsHap v.1.0, ShapeIt4 v.4.1.3, ShinyGo v.0.8. All custom scripts used in this study can be retrieved from: https://github.com/hugocrvl/TytoAlbaLocalAdaptation.

For manuscripts utilizing custom algorithms or software that are central to the research but not yet described in published literature, software must be made available to editors and reviewers. We strongly encourage code deposition in a community repository (e.g. GitHub). See the Nature Portfolio guidelines for submitting code & software for further information.

Data

Policy information about availability of data

All manuscripts must include a data availability statement. This statement should provide the following information, where applicable:

- Accession codes, unique identifiers, or web links for publicly available datasets
- A description of any restrictions on data availability
- For clinical datasets or third party data, please ensure that the statement adheres to our policy

The data underlying this article are available in the GenBank Sequence Read Archive Database at https://www.ncbi.nlm.nih.gov/sra, and can be accessed within Bioproject PRJNA700797, Bioproject PRJNA727915 and Bioproject PRJNA727977.

Research involving human participants, their data, or biological material

Policy information about studies with human participants or human data. See also policy information about sex, gender (identity/presentation), and sexual orientation and race, ethnicity and racism.

Reporting on sex and gender	N/A
Reporting on race, ethnicity, or other socially relevant groupings	N/A
Population characteristics	N/A
Recruitment	N/A
Ethics oversight	N/A

Note that full information on the approval of the study protocol must also be provided in the manuscript.

Field-specific reporting

Please select the one below that is the best fit for your research. If you are not sure, read the appropriate sections before making your selection.

☐ Life sciences ☐ Behavioural & social sciences ☒ Ecological, evolutionary & environmental sciences

For a reference copy of the document with all sections, see nature.com/documents/nr-reporting-summary-flat.pdf

Ecological, evolutionary & environmental sciences study design

All studies must disclose on these points even when the disclosure is negative.

Study description	In this study, we combined ecological modelling with genomic analyses to investigate the spatiotemporal patterns and mechanisms of local adaptation of the Western Palearctic barn owl (Tyto alba).
Research sample	Publicly available samples from Cumer et al. (2022), Machado, Cumer, et al. (2021), and Machado, Topaloudis, et al. (2021).
Sampling strategy	We used all available continental samples.
Data collection	We retrieved resequencing samples from GenBank, see "Data" section for accession code.
Timing and spatial scale	Cumer et al. (2022), Machado, Cumer, et al. (2021), and Machado, Topaloudis, et al. (2021).
Data exclusions	One sample from Switzerland was not retrieved from GenBank because of its low phasing quality.
Reproducibility	All resequencing data, reference genome and the custom scripts are publicly available (stated in the manuscript).
Randomization	Randomisation is not relevant for this study. Coordinates of sampling were reported in Supplementary Material.
Blinding	Blinding is not relevant for this study.

Did the study involve field work? ☐ Yes ☒ No

Reporting for specific materials, systems and methods

We require information from authors about some types of materials, experimental systems and methods used in many studies. Here, indicate whether each material, system or method listed is relevant to your study. If you are not sure if a list item applies to your research, read the appropriate section before selecting a response.

Materials & experimental systems

n/a	Involved in the study
☒	☐ Antibodies
☒	☐ Eukaryotic cell lines
☒	☐ Palaeontology and archaeology
☒	☐ Animals and other organisms
☒	☐ Clinical data
☒	☐ Dual use research of concern
☒	☐ Plants

Methods

n/a	Involved in the study
☒	☐ ChIP-seq
☒	☐ Flow cytometry
☒	☐ MRI-based neuroimaging

Plants

Seed stocks	N/A
Novel plant genotypes	N/A
Authentication	N/A
